# Drug–drug interaction prediction: databases, web servers and computational models

**DOI:** 10.1093/bib/bbad445

**Published:** 2023-12-18

**Authors:** Yan Zhao, Jun Yin, Li Zhang, Yong Zhang, Xing Chen

**Affiliations:** School of Information and Control Engineering, China University of Mining and Technology, Xuzhou 221116, China; School of Information and Control Engineering, China University of Mining and Technology, Xuzhou 221116, China; School of Information and Control Engineering, China University of Mining and Technology, Xuzhou 221116, China; School of Information and Control Engineering, China University of Mining and Technology, Xuzhou 221116, China; School of Science, Jiangnan University, Wuxi 214122, China

**Keywords:** drug, drug–drug interaction, database, web server, computational model

## Abstract

In clinical treatment, two or more drugs (i.e. drug combination) are simultaneously or successively used for therapy with the purpose of primarily enhancing the therapeutic efficacy or reducing drug side effects. However, inappropriate drug combination may not only fail to improve efficacy, but even lead to adverse reactions. Therefore, according to the basic principle of improving the efficacy and/or reducing adverse reactions, we should study drug–drug interactions (DDIs) comprehensively and thoroughly so as to reasonably use drug combination. In this review, we first introduced the basic conception and classification of DDIs. Further, some important publicly available databases and web servers about experimentally verified or predicted DDIs were briefly described. As an effective auxiliary tool, computational models for predicting DDIs can not only save the cost of biological experiments, but also provide relevant guidance for combination therapy to some extent. Therefore, we summarized three types of prediction models (including traditional machine learning-based models, deep learning-based models and score function-based models) proposed during recent years and discussed the advantages as well as limitations of them. Besides, we pointed out the problems that need to be solved in the future research of DDIs prediction and provided corresponding suggestions.

## Drug

In pharmacology, as a class of chemical substance with known structure, drugs can produce biological effect when they are administered to a living organism [1]. More specifically, a pharmaceutical drug, also known as a medication or medicine, is a chemical substance used to prevent or treat diseases [1]. Unlike food, for patients with different diseases, drugs may be taken into the bodies in different ways, such as inhalation, injection, ingestion, skin application, sublingual dissolution and so on [2]. Besides, in clinical treatment, patients usually take drugs for a limited period of time or periodically over a long period of time [3]. With the development of science and technology, the way of manufacturing drugs has changed a lot [4]. Traditionally, drugs are derived from medicinal plants, but they have also been synthesized organically in recent years [5]. As the number of drugs increase, a number of relevant databases have been constructed for further research [6, 7]. For example, the latest release of DrugBank [6] (version 5.1.10, released 4 January 2023) contains 15 451 drug entries including 2740 approved small molecule drugs, 1577 approved biologics, 134 nutraceuticals and over 6717 experimental drugs.

### Drug–drug interaction

In clinical treatment, to cure or ameliorate symptoms of diseases or medical condition, two or more kinds of drugs are usually needed [8]. The fundamental reason of drug combination lies in the evidence that combination therapy tends to have higher cure rates than monotherapy [9]. For instance, the combination therapy of doxorubicin, cyclophosphamide, vincristine and prednisone is commonly implemented in cancer chemotherapy regimens [10, 11]. Another example is that the combined application of anti-tuberculosis drugs not only enhances the drug efficacy but also delays the emergence of drug resistance of *Mycobacterium tuberculosis*, which is the main cause of tuberculosis [12]. However, the risk of harmful drug–drug interactions (DDIs) increases as patients take more kinds of drugs [13]. For example, more than one-third of older Americans regularly use five or more drugs or supplements, and 15% are at risk for serious DDIs [14]. Specifically, DDIs refer to the interactions between drugs used in drug therapy or between drugs and metabolites, endogenous substances, food and diagnostic agents [15]. DDIs can either enhance the efficacy (synergistic action) or decrease the efficacy (antagonistic action) by resulting in changes in the nature, intensity, duration, side effects and toxicity of drugs [16]. In other words, the consequences of DDIs include the desired, the insignificant (vast majority) and the harmful reaction [17]. In general, we are more concerned about harmful DDIs in terms of drug safety. To improve drug safety, it is necessary to fully understand the pharmacological action of each drug before combination therapy so as to achieve the best curative effect and the least adverse drug reactions [18].

Drugs in combination prescriptions would influence each other’s effects and change the way they work in the human body [19]. This kind of influence can be divided into pharmaceutical interactions, pharmacodynamics (PD) interactions and pharmacokinetics (PK) interactions [19]. Pharmaceutical interactions refer to the change of drug action caused by chemical reaction due to unreasonable dispensing, that is, interactions *in vitro* before drugs enter the body [20]. An example of pharmaceutical interaction is that tetracycline and calcium salt injection may result in precipitate due to the formation of chelate under neutral or alkaline conditions [21]. PD interaction means that two drugs share the same receptor, and one drug has antagonistic, additive, synergistic or indirect pharmacological effects on the other drug [13]. For instance, because both atropine and tubocurarine reversibly bind to receptors, the combination therapy of these two drugs blocks the action of the normal physiological transmitter acetylcholine [22]. PK interactions refer to the interference caused by simultaneous or sequential use of two or more drugs in the metabolic stage (absorption, distribution, metabolism and elimination), resulting in enhanced efficacy or adverse reactions [23]. Besides, different from PD interactions, PK interactions often lead to changes in the blood concentration of the interacting drugs [23]. For example, the combination therapy of warfarin and nonsteroidal anti-inflammatory drugs would result in a PK interaction [24]. In detail, some nonsteroidal anti-inflammatory drugs can inhibit warfarin metabolism, thereby enhancing the effect of warfarin on hypoprothrombinemia and significantly increasing the risk of bleeding [24]. For those patients who are receiving combination therapy, unidentified DDIs may reduce efficacy, cause unexpected side effects or other adverse drug reactions and even endanger life [25]. The harmful reactions from DDIs include bleeding, bone marrow suppression, arrhythmias, hypotension, rhabdomyolysis, central nervous system depression, seizures, hypoglycemia, renal failure and so on [26].

Hence, paying attention to DDIs is of great significance to adopt effective combination therapy and further improve the quality of medical treatment. Moreover, in-depth understanding of the absorption, distribution, metabolism and excretion process of drugs in the body, as well as the interactions between various drugs in the body, can reduce adverse drug reactions and ensure drug safety [27]. Nevertheless, as a new drug is approved, the potential DDIs resulting from different drug combinations will grow exponentially. In this case, it is not hard to imagine that the process of validating potential DDIs one by one through biological experiments is very expensive and time-consuming [28]. With the rapid development of computing technology, large-scale DDIs analysis and prediction have also made great strides. In the face of the huge number of existing available drugs, computational models can be used to screen drug combinations with high probability of interaction [16].

As increasing evidence show that the discovery of potential DDIs plays an important role in drug development and disease treatment, more and more researchers devote themselves to corresponding studies. In the following parts, we first reviewed the existing databases and web servers about DDIs. Then, we introduced three types of computational models and discussed their advantages and disadvantages. Furthermore, we put forward the problems that need to be solved in the future research of DDIs prediction and provided corresponding suggestions.

### Databases and web servers

With the rapid development of drug-related research studies, more and more drug-related databases and web servers have been constructed to facilitate researchers to carry out deeper research studies. Next, we briefly introduced some representative databases as well as web servers and summarized them in Table 1.

**Table 1:** The function and URL of databases as well as web servers

Databases or web servers	Function	URL
DrugBank	Recording 15 451 drugs and providing more than 200 data fields for each drug, with half of the information devoted to chemical, pharmacological, pharmaceutical and other aspects of the drug and the other half dedicated to documenting the sequence, structure and pathway of the drug target.	https://www.drugbank.ca/
DDInter	Recording 236 834 DDIs involving 1833 drugs and documenting the detailed information about each DDI, such as mechanisms, risk levels, recommendations for drug adjustment and so on.	http://ddinter.scbdd.com
SuperDRUG2	Recording the annotation of drugs, including regulatory details, chemical structures (2D and 3D), dosage, biological targets, physicochemical properties, external identifiers, side-effects, pharmacokinetic data and DDIs.	http://cheminfo.charite.de/superdrug2
INXBASE	Recording more than 20 000 DDIs	https://www.medbase.fi/en/professionals/inxbase/
OncoRx	Documenting 943 DDIs between 117 ACDs and 166 CAMs.	http://www.onco-informatics.com/oncorx/index.php
DIDB	Recording the drug interaction results derived from drug–drug, drug–food and drug–herb interaction studies.	https://www.druginteractionsolutions.org/
DrugComb	Documenting the standardized results of drug combination screening studies about 739 964 combinations involving 8397 drugs.	https://drugcomb.fimm.fi/
DailyMed	Recording the essential scientific information for the safe and effective use of the drugs, such as indications, dosage, administration, adverse reactions, DDIs and so on.	https://dailymed.nlm.nih.gov/dailymed/about-dailymed.cfm
PolySearch2	Predicting the relationships between biomedical entities, such as human diseases, genes, SNPs, proteins, drugs, metabolites and so on.	http://polysearch.ca
DDI-CPI	Presenting the predicted probabilities of interactions between the given drug and 2515 drugs in the library of DDI-CPI.	http://cpi.bio-x.cn/ddi/
vNN-ADMET	Predicting the ADMET properties of drugs.	https://vnnadmet.bhsai.org/vnnadmet/login.xhtml

**Table 2:** The significance and related link of computational models

Model	Significance	Link to the GitHub or sites
Bayesian probabilistic method-based model [13]	Introducing the system connection score and drug phenotypic similarity score	http://www.picb.ac.cn/hanlab/DDI
INDI [57]	Applying a novel scoring scheme to construct the feature vectors of drug pairs based on multiple types of drug similarity	
Label propagation-based model [65]	Implementing label propagation based on multiple similarity information	
Collective PSL-based model [71]	Applying the hinge-loss MRFs to identify potential DDIs in the multigraph through maximum a posteriori	
Random forest-based model [73]	Introducing the enrichment score of the targets of drugs	
Logistic regression-based model [81]	Implementing prediction based on two interaction networks constructed based on the information about PK and PD interactions	
PUL-based model [84]	Applying the growing self- organizing maps clustering algorithm to identify reliable negative samples	
Meta-learning-based model [88]	Using node2vec to get the feature vectors of drugs from the feature network	
MRMF [93]	Introducing manifold regularization into matrix factorization	
DDINMF [96]	Introducing the feature matrix of drug into matrix factorization to make the model suitable for predicting enhancive and degressive DDIs between known drugs and new drugs	
TMFUF [99]	Being suitable for predicting not only known but also new drugs that interact with new drugs	
LCM-DS [100]	Introducing the Dempster–Shafer theory of evidence to integrate the results of three local classification models	https://github.com/JustinShi2016/ScientificReports2018
DDIGIP [106]	Applying the KNNs to fill in the adjacency matrix	
Gradient boosting-based model [107]	Using the TPE approach to optimize the hyperparameters of the classifier	
Network algorithm and matrix perturbation algorithm-based model [114]	Applying the classifier ensemble rule to take the logistic regression to map the outputs of all models to a score as the final prediction result	https://github.com/zw9977129/drug-drug-interaction/
HNAI [118]	Applying five prediction models to identify potential DDIs, respectively	
IAC [121]	Introducing the action crossing method to obtain the feature vectors of drug pairs according to the information about drug–enzyme and drug–transporter actions	
SFLLN [122]	Introducing the sparse feature learning ensemble method to project drugs from different feature spaces to the common interaction space	https://github.com/BioMedicalBigDataMiningLabWhu/SFLLN
DDIMDL [128]	Applying the DNN to calculate the interaction probabilities based on the feature vectors of drugs	https://github.com/YifanDengWHU/DDIMDL
SSI-DDI [129]	Applying the GAT layers to extract the feature vectors of atoms contained in drugs	https://github.com/kanz76/SSI-DDI
STNN-DDI [132]	Introducing tensor to describe the interactions between substructures of drugs	https://github.com/zsy-9/STNN-DDI
META-DDIE [135]	Introducing the chemical sequential pattern mining algorithm to obtain a set of discrete frequent substructures of drugs	https://github.com/YifanDengWHU/META-DDIE
DANN-DDI [139]	Introducing the structural deep network embedding method to learn the embeddings of drugs from interaction networks	https://github.com/naodandandan/ DANN-DDI
MRCGNN [143]	Introducing the contrastive learning to obtain the representations of drugs	https://github.com/Zhankun-Xiong/MRCGNN
MCFF-MTDDI [146]	Introducing the extra label-based feature vector to make the model suitable for multi-label prediction	https://github.com/ChendiHan111/MCFF-MTDDI
DSIL-DDI [149]	Introducing the GNN to extract the substructure representations of drugs	
DSN-DDI [151]	Applying the intra-view and inter-view representation learning methods to obtain the representations of drugs	https://github.com/microsoft/Drug-Interaction-Research/tree/DSN-DDI-for-DDI-Prediction
BioDKG-DDI [154]	Applying a novel similarity fusion method to fuse multiple similarity matrixes of drugs	
MDF-SA-DDI [159]	Introducing the multi-head self-attention mechanism to integrate the feature vectors of each drug pair	https://github.com/ShenggengLin/MDF-SA-DDI
Deep feed- forward network-based model [161]	Introducing the GO term-based drug similarity	
R^2^-DDI [164]	Applying the MLP to obtain the refinement vectors of drugs	https://github.com/linjc16/R2-DDI
Graph kernel-based approach [167]	Constructing all-path graph kernels to describe the connections between syntactic and semantic within the sentences	https://sbmi.uth.edu/ccb/resources/ddi.htm
Semantic predication-based model [174]	Introducing four types of semantic predication generated by SemRep	
Att-BLSTM [177]	Combining attention mechanism and the RNN with BLSTM to learn the global semantic representation of the sentence	
PM-BLSTM [179]	Applying a rule to filter the drugs to ensure that only one drug pair in each sentence was studied	
A two-stage DDIs extraction model [181]	Applying the SVM classifier to identify DDIs and the LSTM-based classifier to identify the type of DDIs	
IK-DDI [186]	Introducing key external text derived from the DrugBank	https://github.com/DouMingLiang/IK-DDI
3DGT-DDI [189]	Introducing the 3D structure conformations of drugs	https://github.com/hehh77/3DGT-DDI
Russell–Rao-based model [192]	Applying the Russell–Rao method to calculate interaction probability	
Score matrix and PCA-based model [194]	Applying PCA method to integrate the score matrixes to obtain the interaction probability matrix	

### Databases

We here briefly introduced several well-known databases that were built to store various data related to DDIs such as DrugBank [6], DDInter [29], SuperDRUG2 [30], INXBASE [31], OncoRx [32], DIDB [33], DrugComb [7] and DailyMed. Except for DDIs, users can also retrieve many other useful information about drugs from these databases, including drug target, metabolic pathways, crystal structures, regulatory details, indications, side-effects, physicochemical properties, pharmacokinetics and so on.

#### DrugBank (https://www.drugbank.ca/)

The latest release of DrugBank (version 5.1.10, released on 4 January 2023) contains 15 451 drugs including 2740 approved small molecules, 1577 approved biologics (proteins, peptides, vaccines and allergenics), 134 nutraceuticals and over 6717 experimental drugs [6]. Each drug in DrugBank contains more than 200 data fields with half of the information being devoted to introducing drugs from the aspects of chemistry, pharmacology as well as pharmacy, and the other half being devoted to recording the sequence, structure and pathway of the drug target. In addition, DrugBank records more than 1.3 million DDIs and provides DDIs Checker, through which users can check the interactions between up to five drugs at one time.

#### D‌DInter (http://ddinter.scbdd.com)

As a comprehensive database dedicated to DDI research, the DDInter not only records 236 834 DDIs involving 1833 drugs but also documents the detailed information about each DDI, such as mechanisms, risk levels, recommendations for drug adjustment and so on [29]. Besides, similar to DrugBank, DDInter also provides Interaction Checker for users to check whether drugs interact with each other.

#### SuperDRUG2 (http://cheminfo.charite.de/superdrug2)

The SuperDRUG2 database is intended to serve as a comprehensive knowledge base of approved and marketed 4587 drugs (involving small molecule, biological products and other drugs) [30]. The annotation of drugs contains regulatory details, chemical structures (2D and 3D), dosage, biological targets, physicochemical properties, external identifiers, side-effects, pharmacokinetic data and DDIs. Besides, SuperDRUG2 can be used to infer potential DDIs and further provide alternative recommendations for elderly patients.

### INXBASE (https://www.medbase.fi/en/professionals/inxbase/)

INXBASE (formerly named SFINX), a database that records more than 20 000 DDIs, can be easily integrated to health information systems and accessible through a portal, which helps the healthcare professionals choose the most appropriate action to overcome specific DDIs [31]. The database has become a basic tool to avoid DDIs for physicians, pharmacists or nurses. Also, a patient-oriented version is available.

#### OncoRx (http://www.onco-informatics.com/oncorx/index.php)

OncoRx is an oncology database that documents 943 DDIs between 117 anticancer drugs (ACDs) and 166 complementary and alternative medicines (CAMs) [32]. What needs to be pointed out is that OncoRx primarily covers PK and PD DDIs, as these two kinds of interactions explain most of the clinically relevant interactions between drugs. Besides, when users applied OncoRx to search DDIs, some important information would also be provided, such as DDIs parameters, pharmacokinetic data on ACDs and CAMs as well as characteristics of CAMs based on traditional Chinese medicines principles.

#### DIDB (https://www.druginteractionsolutions.org/)

The Drug Interaction Database (DIDB) is designed to support the decision-making process of scientists in evaluating PK DDIs and drug safety, which is composed of human *in vitro* and *in vivo* datasets [33]. The human *in vitro* datasets contain results from both metabolism and transporter studies, while the human *in vivo* datasets include the studies result about organ impairment, pharmacogenetics and drug interaction, where the drug interaction results are derived from drug–drug, drug–food and drug–herb interaction studies.

### DrugComb (https://drugcomb.fimm.fi/)

As an open-access data portal, DrugComb documents the standardized results of drug combination screening studies about 739 964 combinations involving 8397 drugs [7]. In addition, DrugComb provides a web server, through which users can analyze and visualize their own drug combination screening data.

#### DailyMed (https://dailymed.nlm.nih.gov/dailymed/about-dailymed.cfm)

The DailyMed database contains labels for two types of drugs, namely, FDA-approved drugs (such as prescription drug, nonprescription drug, certain medical devices, etc.) and additional drugs regulated but not approved by the FDA (such as dietary supplements and unapproved prescription as well as nonprescription, etc.). It should be pointed out that the labels of prescription drug and biological products contain a summary of the essential scientific information for the safe and effective use of the product, such as indications, dosage, administration, adverse reactions, DDIs and so on.

### Web servers

Except for databases, there are also some online web servers that can be used to analyze or predict DDIs, such as PolySearch2 [34], DDI-CPI [35] and vNN-ADMET [36].

#### PolySearch2 (http://polysearch.ca)

PolySearch2, an online text-mining system, can provide relationships between biomedical entities, such as human diseases, genes, single-nucleotide polymorphisms (SNPs), proteins, drugs, metabolites and so on [34]. Specifically, for one given entity that support retrieval, PolySearch2 will return all types of aforementioned entities associated with this entity. In the search results, each type of entity is sorted in reverse order based on the *Z*-score calculated by PolySearch [37]. Synonyms of each entity as well as key sentences mined from literatures to confirm the corresponding association are also provided. It should be pointed out that, to improve the accuracy and coverage, the retrieval results presented to users are mined from well-known free-text collections (e.g. MEDLINE, PubMed and Wikipedia) and biological databases (e.g. UniProt and DrugBank).

#### D‌DI-CPI (http://cpi.bio-x.cn/ddi/)

Considering that a large amount of DDIs are mediated by drug–protein interactions, the DDI-CPI server [35] is constructed to predict potential DDIs based on the chemical–protein interactome (CPI), which is a methodology that mimics the theoretical interactions between drug and proteins using silicon simulations [38]. For a given drug, DDI-CPI will present the predicted probabilities of interactions between the drug and 2515 drugs in the library of DDI-CPI.

#### vNN-ADMET (https://vnnadmet.bhsai.org/vnnadmet/login.xhtml)

Through the vNN-ADMET webserver [36], users can obtain the absorption, distribution, metabolism, excretion and toxicity (ADMET) properties of drugs by using one of the fifteen models constructed based on the variable nearest neighbor (vNN) method [39]. For example, these models could be applied to predict some important properties of the given drug, such as cytotoxicity, mutagenicity, cardiotoxicity, DDIs, microsomal stability and drug-induced liver injury.

### Computational models

As mentioned above, detecting DDIs is beneficial to clinical drug combination treatment. However, due to the high cost and long cycle of experimental methods, it is of great significance to develop effective computational models to infer potential DDIs on a large scale. During recent years, to predict unknown DDIs, researchers have built a number of computational models, which could be divided into three categories: traditional machine learning-based models, deep learning-based models and score function-based models.

### Traditional machine learning-based models

Traditional machine learning algorithms have been widely used to solve complex problems in industrial application [40, 41] and biological science [42–52]. Here, traditional machine learning-based prediction models mainly covers label propagation, Markov random fields (MRFs), random forest, logistic regression, support vector machine (SVM), matrix factorization, ensemble learning and so on. Traditional machine learning-based models could be used to predict DDIs on a large scale and are suitable for new drugs. However, there are still some limitations to be resolved. For example, in the models constructed based on supervised learning algorithms, unlabeled samples are treated as negative samples because of the lack of highly reliable negative samples. Besides, for the parameters involved in the traditional machine learning-based models, researchers often randomly set the values of the parameters rather than using some algorithms to obtain the optimal values of the parameters, which limits the performance of models to some extent. Moreover, researchers tended to obtain the feature vectors of drug pairs by splice the feature vectors of corresponding drugs directly, so constructing more significant feature vectors is still an urgent problem to be solved.

#### Bayesian probabilistic method-based model

**Figure 1:**
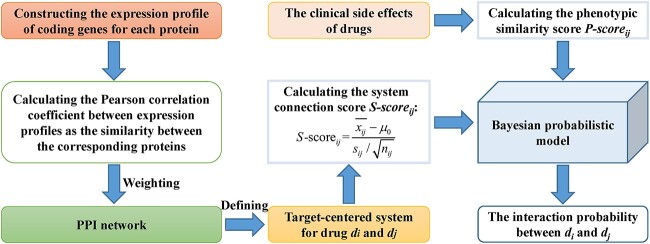
The flowchart of Bayesian probabilistic method-based model, where the interaction probabilities between drugs are calculated by integrating the system connection score and phenotypic similarity score through a Bayesian probabilistic method.

Based on the hypothesis that the smaller the minimum distance between the targets of two drugs in the PPI network constructed based on the Human Protein Reference Database [53], the greater the possibility of PD interaction between the corresponding drugs, Huang *et al*. [13] designed a model for PD interaction prediction by considering drug actions in the PPI network (Figure 1). Firstly, for protein *p_i_*, the authors constructed its coding gene’s expression profile across 79 human tissues [54], denoted by *EP_i_* (79D vector). Secondly, for the protein pair (*p_i_,p_j_*) with known interaction, the authors calculated the Pearson correlation coefficient between *EP_i_* and *EP_j_* to weight the edge connecting *p_i_* and *p_j_* in the PPI network. Besides, for each drug, the authors constructed a target-centered system consisting of the target proteins of the corresponding drug and the first-step neighboring proteins of the target protein in the PPI network. Finally, for drugs *d_i_* and *d_j_*, the system connection score *S-score_ij_* was calculated to describe the tightness of connection between the target-centered systems of *d_i_* and *d_j_*:


(1) \begin{equation*} S- scor{e}_{ij}=\frac{\overline{x_{ij}}-{\mu}_0}{s_{ij}/\sqrt{n_{ij}}} \end{equation*}

where $\overline{x_{ij}}$ and *s_ij_* represent the mean and SD of edge weights connecting the proteins in the target-centered system of *d_i_* and *d_j_*, respectively; *n_ij_* denotes the number of edges connecting two target-centered systems; and ${\mu}_0$ refers to the mean of all edge weights in the PPI network. It should be noted that if two target-centered systems had a common protein, an artificial edge with a weight of 1 was added between the two systems. In addition, following the previous research [55], the drug phenotypic similarity score *P-score_ij_* between *d_i_* and *d_j_* was calculated based on the clinical side effects of drugs. Finally, inspired by the Bayesian probabilistic model proposed by Xia *et al.* [56], the likelihood ratio (LR) for drug pair (*d_i_,d_j_*) to be true-positive DDIs versus true-negative DDIs based on *S-score_ij_* and *P-score_ij_* were calculated:


(2) \begin{equation*} LR\left(S- scor{e}_{ij}\right)=\frac{P\left(S- scor{e}_{ij}| positive\right)}{P\left(S- scor{e}_{ij}| negative\right)} \end{equation*}

(3) \begin{equation*} LR\left(P- scor{e}_{ij}\right)=\frac{P\left(P- scor{e}_{ij}| positive\right)}{P\left(P- scor{e}_{ij}| negative\right)} \end{equation*}

Then, by multiplying the LRs calculated based on two independent evidences (i.e. *S-score_ij_* and *P-score_ij_*), the interaction probability *P_ij_* between *d_i_* and *d_j_* was obtained:


(4) \begin{equation*} {P}_{ij}= LR\left(S- scor{e}_{ij}\right)\times LR\left(P- scor{e}_{ij}\right) \end{equation*}

#### INDI

Instead of predicting a single type of DDIs, the model of INferring Drug Interactions (INDI) proposed by Gottlieb *et al*. [57] could be used to infer PD, PK and potential PK interactions (the drug pair was metabolized by the same cytochrome P450 enzyme but there was no interaction evidence between these two drugs). Firstly, the authors collected these three types of DDIs from DrugBank [6] and Drugs.com (http://drugs.com). Then, they calculated drug similarity from seven aspects, including the chemical structure [58], receptors [59], side effects [60] and anatomical therapeutic chemical (ATC) codes [61] of drugs, the sequence of drug targets [62], the distances between drug targets on the human PPI network [63] and the semantic similarity between drug targets [61], which were denoted by *S_i_* (*i* = 1,2,….6, 7), respectively. Further, they integrated the above multiple drug–drug similarities to obtain the features of drug pairs. Specifically, given a drug pair (*d*_1_*,d*_2_) without verified interaction, for each known interaction $\left({d}_1^{\prime},{d}_2^{\prime}\right)$, they first computed the drug–drug similarities${S}_i\left({d}_1,{d}_1^{\prime}\right)$,${S}_i\left({d}_2,{d}_2^{\prime}\right)$,${S}_i\left({d}_1,{d}_2^{\prime}\right)$ and ${S}_i\left({d}_2,{d}_1^{\prime}\right)$. Next, according to the scoring scheme proposed in literature [64]*,* they calculated the score *Score*(*d*_1_*,d*_2_) between *d*_1_ and *d*_2_ as follows:


(5) \begin{align*}& {S}_{ij}\left({d}_1,{d}_2,{d}_1^{\prime},{d}_2^{\prime}\right)=\nonumber\\&\max \left(\sqrt{S_i\left({d}_1,{d}_1^{\prime}\right)\times{S}_j\left({d}_2,{d}_2^{\prime}\right)},\sqrt{S_i\left({d}_1,{d}_2^{\prime}\right)\times{S}_j\left({d}_2,{d}_1^{\prime}\right)}\right) \end{align*}

(6) \begin{equation*} Scor{e}_{ij}\left({d}_1,{d}_2\right)=\underset{\begin{array}{l}\left\{{d}_1^{\prime},{d}_2^{\prime}\right\}\cap \left\{{d}_1,{d}_2\right\}=\varnothing \\{}\kern1.5em \left({d}_1^{\prime},{d}_2^{\prime}\right)\in{D}_p\end{array}}{\max }{S}_{ij}\left({d}_1,{d}_2,{d}_1^{\prime},{d}_2^{\prime}\right) \end{equation*}

where *D_p_* represents the set consisting of drug pairs with known interactions; *i,j* = 1,2…6, 7. Therefore, 49 scores [i.e. *Score*_11_(*d*_1_*,d*_2_), *Score*_12_(*d*_1_*,d*_2_)*…Score*_76_(*d*_1_*,d*_2_), *Score*_77_(*d*_1_*,d*_2_)] between *d*_1_ and *d*_2_ could be obtained, which are regarded as the features of the drug pair (*d*_1_, *d*_2_). Finally, a logistic regression classifier was implemented to predict three types of DDIs based on the features of drug pairs.

#### Label propagation-based model

By integrating the information about side effects and chemical structure of drugs, Zhang *et al*. [65] proposed a model to predict potential DDIs based on the label propagation algorithm. Firstly, the authors downloaded the information about the label and off-label side effect of drugs from SIDER [66] and OFFSIDES [67], respectively. Besides, the chemical structure information was extracted from PubChem [68]. Secondly, for each drug, three binary feature vectors were constructed according to the information about label side effect, off-label side effect and chemical structure, respectively. Then, the Jaccard index was used to calculate the similarities between drugs and three corresponding similarity matrices were constructed later. Thirdly, the authors used the Bregmanian Bi-Stochastication (BBS) algorithm [69] to normalize the three similarity matrices and the normalized matrixes were denoted as *W_k_*(*k* = 1–3). Besides, the adjacency matrix *A* was constructed based on the known DDIs, whose row was defined as the label of the corresponding drug. As for label propagation, in the *t*th iteration, the drug nodes absorbed the label information of the neighbor nodes at a ratio of $\mu$ and retained the original label information with a ratio of $\left(1-\mu \right)$ to update the label. Therefore, based on the *k*th similarity matrix *W_k_*, the interaction probability matrix ${P}_k^t$ could be obtained as follows:


(7) \begin{equation*} {P}_k^t=\mu{W}_k{P}_k^{t-1}+\left(1-\mu \right)A \end{equation*}

where ${P}_k^0=A$. The matrix ${P}_k^t$ obtained after iteration convergence is the final interaction probability matrix, which could also be obtained by minimizing the following objective function:


(8) \begin{equation*} \underset{P_k}{\min}\ \mu tr\left({P}_k^T\left(I-{W}_k\right){P}_k\right)+\left(1-\mu \right){\left\Vert{P}_k-A\right\Vert}_F^2 \end{equation*}

where $tr\left(\right)$ refers to the trace of a matrix; ${\left\Vert \right\Vert}_F$ refers to the Frobenius norm. To introduce multiple similarity information about drugs, the authors calculated the converged solution by solving the following composite optimization problem:


(9) \begin{equation*} {\displaystyle \begin{array}{l}\underset{P,\alpha }{\min \mu \mathrm{tr}}\left({P}^T\sum \limits_{k=1}^3{\alpha}_k\left(I-{W}_k\right)P\right)+\left(1-\mu \right){\left\Vert P-A\right\Vert}_F^2+\delta{\left\Vert \alpha \right\Vert}_2^2\ \\{}\mathrm{s}.\mathrm{t}.,\forall k,{\alpha}_k\ge 0,\sum \limits_{k=1}^3{\alpha}_k=1\end{array}} \end{equation*}

where $\delta$ refers to the regularization parameter; $\alpha =\left[{\alpha}_1,{\alpha}_2,{\alpha}_3\right]$ represents the vector composed of weight coefficients; ${\left\Vert \right\Vert}_2$ represents the Euclidean norm. Finally, the block coordinate descent (BCD) [70] schema was applied to calculated *P* and $\alpha$, where the element of matrix *P* was regarded as the interaction probability between the corresponding two drugs.

#### Collective probabilistic soft logic-based model

Through the collective probabilistic soft logic (PSL) framework, Sridhar *et al.* [71] inferred potential DDIs based on multiple drug similarities and known DDIs. Specifically, the authors calculated seven kinds of drug similarity, including chemical structure-based [58], ligand-based [59], side effect-based [60], drug annotation-based [61], target protein sequence-based [62], PPI network-based [63] and Gene Ontology (GO)-based [61] drug similarities. Besides, by representing drugs with nodes, the authors constructed a multigraph with eight types of edges (denoting the above seven types of drug similarity and known DDIs, respectively). Then, first order logic-syntax was used by PSL to template for a special class of MRFs models called hinge-loss MRFs (HL-MRFs). Finally, HL-MRFs was applied to identify potential DDIs in the multigraph through maximum a posteriori (MAP) [72] based on the rule that if drug *d_i_* and *d_j_* were similar, and there was known DDI between *d_j_* and *d_k_*, *d_i_* was likely to interact with *d_k_*.

#### Random forest-based model

Liu *et al*. [73] proposed a random forest-based method to predict unknown DDIs. Before training the random forest model, each drug pair was represented by a feature vector derived from three aspects: chemical interaction between drugs [74], protein interactions between the targets of drugs and enrichment score of the targets of drugs. Taking the drug pair (*d_i_,d_j_*) as an example, the feature based on the chemical interaction referred to the ‘Combined_score’ of the drug pair recorded in STITCH [75]. According to the protein interaction score documented in STRING [76], the features of the drug pairs were defined based on different target protein sets and the same target protein set, respectively. Specifically, the author took the maximum $D{S}_{ij}^m$ and average value $D{S}_{ij}^a$ of the interaction scores between the target proteins of *d_i_* and the target proteins of *d_j_* as the features built based on a different target protein set. As for the features based on the same target protein set, for these proteins in the target protein set of drug *d_i_*, the authors calculated the maximum $S{S}_i^m$ and average value $S{S}_i^a$ of the interaction scores between these proteins. In the same way, the maximum $S{S}_j^m$ and average value $S{S}_j^a$ of the interaction scores between the target proteins of the drug *d_j_* were obtained. Then, four features of the drug pair (*d_i_,d_j_*) were defined (i.e. $S{S}_i^m+S{S}_j^m,S{S}_i^a+S{S}_j^a,\left|S{S}_i^m-S{S}_j^m\right|,\left|S{S}_i^a-S{S}_j^a\right|$). The authors also constructed the feature vectors of drug pairs based on the enrichment score of target proteins in 229 pathways in Kyoto Encyclopedia of Genes and Genomes (KEGG) [77]. Specifically, for drugs *d_i_* and *d_j_*, the authors calculated their target enrichment scores in these pathways, respectively, denoted by $e{s}_i^1,e{s}_i^2,\dots, e{s}_i^{229},e{s}_j^1,e{s}_j^2,\dots, e{s}_j^{229}$. Then, according to the enrichment scores, 458 features of drug pair (*d_i_,d_j_*) were defined (i.e. $e{s}_i^1+e{s}_j^1,e{s}_i^2+e{s}_j^2,\dots, e{s}_i^{229}+e{s}_j^{229},\kern0.33em \left|e{s}_i^1-e{s}_j^1\right|,\left|e{s}_i^2-e{s}_j^2\right|,\dots, \left|e{s}_i^{229}-e{s}_j^{229}\right|$). Minimum redundancy maximum relevance [78] as well as incremental feature selection [78] were used to implement feature extraction, and the final 386 features were selected for the drug pair based on the value of the Matthews’s correlation coefficient [79]. Finally, the random forest algorithm with its default configuration (in Weka 3.6.4 [80]) was adopted to train the prediction model.

#### Logistic regression-based model

Based on the assumption that a query drug (Dq) tends to interact with a drug to be examined (De) if Dq is structurally similar to drugs in the interaction network of De, Takeda *et al*. [81] proposed a DDI prediction model highly relying on the 2D structural similarities between Dq and all drugs in the interaction network of De. Firstly, for each De, the authors constructed two interaction networks based on the PK and PD information about it. The nodes in the network constructed based on PK information represented enzymes as well as transfer proteins associated with De and drugs associated with the above-mentioned enzymes as well as transfer proteins. It should be pointed out that the drugs in the network could be divided into three categories, that is, drugs that interact with enzymes, drugs that have pharmacogenetic associations with enzymes and drugs that are transported by transfer proteins. While the nodes in the network constructed based on PD information refer to the target protein of De, the drugs associated with the target protein, other proteins that interact with the target protein and the drugs associated with these proteins. Similarly, the drugs in the network are also divided into three categories, namely, drugs that target the target protein, drugs that have pharmacogenetic association with the target protein and drugs having pharmacogenetic association with the protein interacted with target proteins. Secondly, according to the PubChem 2D fingerprint [82] and the Tanimoto coefficient, the structural similarities between Dq and all the drugs (including De) in the two networks of De were computed. Then, they calculated the maximum similarity between Dq and each type of drugs, respectively, which constituted the feature vector (seven-dimensions) of drug pair (Dq,De) together with the similarity between Dq and De. After constructing the balanced classification dataset [the number of (Dq,De) pairs with known interactions is the same as the number of pairs without interactions], the authors trained the logistic regression model by using the generalized linear models (glm) implemented in R package *caret* based on the feature vectors of drug pairs [83]. Finally, they applied the trained logistic regression model to predict the potential DDIs.

#### Positive-unlabeled learning-based model

To deal with the problem of rarely available negative samples, Hameed *et al.* [84] proposed a positive-unlabeled learning (PUL) method to infer potential DDIs. Initially, based on the chemical structure [6], indication [85], target protein [6] and side effect [86] information of drugs, four binary vectors ${f}_k\left(k=1,2,3,4\right)$ were constructed as the feature vectors of the corresponding drug, respectively. Then, based on these feature vectors, two types of feature vectors of drug pairs were defined. Specifically, the first type was Jaccard index-based similarity feature representation 1 (SFR1), where the drug similarity matrix ${S}_k\left(k=1,2,3,4\right)$ was calculated based on the *k*th feature vector using the Jaccard index. Therefore, according to SFR1, the author could obtain the 4D feature vector ${F}_1^{ij}$ of drug pair (*d_i_*, *d_j_*):


(10) \begin{equation*} {F}_1^{ij}=\left({S}_1\left({d}_i,{d}_j\right),{S}_2\left({d}_i,{d}_j\right),{S}_3\left({d}_i,{d}_j\right),{S}_4\left({d}_i,{d}_j\right)\right) \end{equation*}

Besides, the authors raised the similarity feature representation 2 (SFR2) to capture the shared properties of drugs. Specifically, the author averaged the corresponding element values of the *k*th feature vectors of *d_i_* and *d_j_*, respectively, to obtain the vector ${F}_{2k}^{ij}$. Then, the authors defined the feature vector ${F}_2^{ij}$ constructed based on SFR2:


(11) \begin{equation*} {F}_2^{ij}=\left({F}_{21}^{ij},{F}_{22}^{ij},{F}_{23}^{ij},{F}_{24}^{ij}\right) \end{equation*}

Secondly, the authors considered 6036 drug pairs with interactions recorded in DrugBank as positive samples, and another 6036 samples were randomly selected from unlabeled samples as candidate samples. Then, they utilized the growing self-organizing maps (GSOM) clustering algorithm [87] to cluster the above samples based on SFR1. If a cluster contained only candidate samples, these candidate samples were regarded as negative samples. In a similar way, negative samples were inferred based on SFR2, and the 589 common negative samples inferred based on SFR1 and SFR2 were considered as the final negative samples. Thirdly, they randomly selected 589 samples from the positive samples as the final positive samples, which were combined with the inferred negative samples to form the training set. The authors repeated the sampling 10 times to construct 10 balanced training sets. Then, the authors applied these training sets to train SVM classifiers based on SFR1 and SFR2, respectively. Finally, the prediction results of the trained 20 classifiers were averaged to obtain the final prediction result.

#### Meta-learning-based model

**Figure 2:**
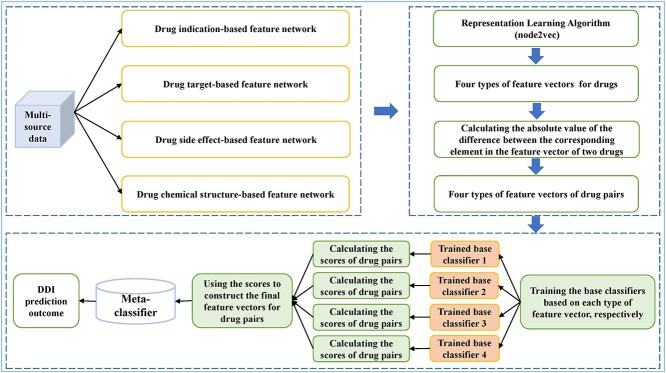
The flowchart of the meta-learning-based model built based on representation learning, PUL and meta-learning.

Similar to the above PUL-based approach, considering that it is difficult to obtain reliable negative samples, Deepika *et al*. [88] proposed a semi-supervised learning framework (Figure 2) for predicting DDIs through combining representation learning [89], PUL [90] and meta-learning [91]. Specifically, the authors first applied the same four types of drug features as the above PUL-based approach to construct corresponding feature network, respectively. Taking the feature network constructed based on the chemical structure as an example, there were two kinds of nodes in the network: drug nodes and chemical substructure nodes. If the drug has a certain substructure, the corresponding two nodes are connected by an edge; otherwise, they are not connected, and the other three feature networks were constructed in a similar way. For each type of feature, drugs in the feature network were represented with a *d*-dimensional feature vector via node2vec [92], which was a representation learning algorithm that was applied to explore the neighborhood information of nodes in the network through biased random walk and then obtain the features of the nodes. For the drug pair (*d*_1_,*d*_2_), a *d*-dimensional vector composed of the absolute value of the difference between the corresponding element in the feature vector of *d*_1_ and *d*_2_ was defined as the feature vector of the drug pair. Next, the authors trained the base classifiers based on each type of feature vector and calculated the weight of each base classifier by cross validation, respectively. Then, the multiplication of the interaction probability predicted by the base classifier and the weight of the classifier was defined as the score of corresponding drug pair. Therefore, based on the four types of features, four scores could be obtained. Then, the final feature vectors (four-dimension) of drug pairs were constructed based on their scores to train the meta-classifier to predict drug pairs with potential interaction from unlabeled samples. It should be pointed out that the bagging SVM classifier constructed based on PUL method was used as the base classifier and meta-classifier of this model.

#### Manifold regularized matrix factorization

Zhang *et al.* [93] presented a novel computational method named manifold regularized matrix factorization (MRMF) to predict potential DDIs by introducing the drug feature-based manifold regularization into the matrix factorization. The authors defined the adjacency matrix *A* based on the known DDIs, where if the drug *d_i_* and *d_j_* have known interaction, then *A_ij_ = 1*, otherwise *A_ij_ = 0*. Then, eight feature vectors of drugs were constructed based on the information about the substructures, targets, enzymes, transporters, pathways, indications, side effects and off side effects of drugs, respectively. Further, the Jaccard similarity matrix (*S*_Jar_), cosine similarity matrix (*S*_Cos_) and Gauss similarity matrix (*S*_Gau_) of drugs were calculated based on the feature vectors, respectively. For matrix factorization, in order to approximate the matrix *A*, two low-rank matrices *X* and *Y* could be obtained by minimizing the following objective function:


(12) \begin{equation*} {\displaystyle \begin{array}{l}L=\frac{1}{2}{\left\Vert A-X{Y}^T\right\Vert}_F^2+\frac{\lambda }{2}\left({\left\Vert X\right\Vert}_F^2+{\left\Vert Y\right\Vert}_F^2\right)\\{}\kern1em =\frac{1}{2}\sum \limits_{ij}{\left({A}_{ij}-{x}_i{y}_j^T\right)}^2+\frac{\lambda }{2}\left(\sum \limits_i{\left\Vert{x}_i\right\Vert}_2^2+\sum \limits_j{\left\Vert{y}_j\right\Vert}_2^2\right)\end{array}} \end{equation*}

where ${\left\Vert A-X{Y}^T\right\Vert}_F^2$ represents the least square cost function, which is used to ensure that the final product of *X* and *Y* approximates the matrix *A*; $\left({\left\Vert X\right\Vert}_F^2+{\left\Vert Y\right\Vert}_F^2\right)$ is used to overcome the overfitting problem; *x_i_* refers to the *i*th row of the matrix *X* and *y_j_* is the *j*th row of the matrix *Y*; $\lambda$ represents the Tikhonov regularization parameter. Given the wide applications of manifold learning [94, 95], the authors treated the similarity between drugs as manifolds and assumed that drugs approximately maintained manifolds in the low-dimensional space. Then, the manifold regularizations for drugs in the low-dimensional space were defined as follows:


(13) \begin{equation*} {L}_{reg}^{row}=\frac{1}{2}\sum \limits_{ij}{\left\Vert{x}_i-{x}_j\right\Vert}_2^2{S}_{ij} \end{equation*}

(14) \begin{equation*} {L}_{reg}^{col}=\frac{1}{2}\sum \limits_{ij}{\left\Vert{y}_i-{y}_j\right\Vert}_2^2{S}_{ij} \end{equation*}

where *S_ij_* is the similarity between drug *d_i_* and *d_j_*. By introducing the manifold regularization, the new objective function was defined as follows:


(15) \begin{equation*} {L}_{new}=L+\frac{\mu }{2}\left({L}_{reg}^{row}+{L}_{reg}^{col}\right) \end{equation*}

where $\mu$ is the manifold regularization parameter. The alternating decent method was applied to minimize the above objective function to obtain the latent feature matrices *X* and *Y*. Then, the interaction probability matrix *P* can be calculated as follows:


(16) \begin{equation*} P=X{Y}^T \end{equation*}

where the element *P_ij_* indicates the interaction probability between drug *d_i_* and *d_j_*.

#### D‌DINMF

Yu *et al*. [96] proposed a semi-nonnegative matrix factorization method to predict enhancive and degressive DDIs (DDINMF), where enhancive (degressive) DDI refers to a drug increases (decreases) the serum concentration of itself and another drug when taken together. The authors considered *m* drugs with known interactions with other drugs as known drugs and *n* drugs without verified interactions with all known drugs as new drugs. In the training phase, different from the definition of adjacency matrix in MRMF, after extracting the enhancive and degressive DDIs information about *m* known drugs, the adjacency matrix *A* was constructed as follows:


(17) \begin{equation*} {A}_{ij}=\left\{\begin{array}{l}1,\kern1em \mathrm{if}\ \mathrm{there}\ \mathrm{is}\ \mathrm{an}\ \mathrm{enhancive}\ \mathrm{drug}\ \mathrm{interaction}\ \mathrm{between}\ {d}_i\ \mathrm{and}\ {d}_j\\{}-1,\mathrm{if}\ \mathrm{there}\ \mathrm{is}\ \mathrm{an}\ \mathrm{degressive}\ \mathrm{drug}\ \mathrm{interaction}\ \mathrm{between}\ {d}_i\ \mathrm{and}\ {d}_j\\{}0,\kern0.75em \mathrm{otherwise}\end{array}\right. \end{equation*}

The authors constructed the *p*-dimensional feature vectors of drugs based on their chemical structure and side effects. Furthermore, the feature vectors of known drugs were combined into feature matrix *F*. Then, two nonnegative low-rank matrices (*W* and *H*) used to approximate matrix *A* could be obtained by minimizing the following objective function:


(18) \begin{equation*} L={\left\Vert A- WH\right\Vert}_F^2 \end{equation*}

After obtaining matrices *W* and *H* by the method proposed by Lee *et al.* [97], to make the model suitable for new drugs, the authors introduced feature matrix into nonnegative matrix factorization. Specifically, they modeled the relationship between the feature matrix *F* and *H* as follows:


(19) \begin{equation*} F\times B={H}^T \end{equation*}

where *B* represents the regression coefficient matrix, which was calculated by SIMPLS algorithm [98]. In the predicting phase, the feature matrix ${F}^{\prime}$ composed of the features of *n* new drugs was mapped into the latent topological space as follows:


(20) \begin{equation*} {H}^{\prime}={\left({F}^{\prime}\times B\right)}^T \end{equation*}

Then, the interaction probabilities between the known drugs and new drugs were calculated as follows:


(21) \begin{equation*} P={\left(W\times{H}^{\prime}\right)}^T \end{equation*}

#### Triple matrix factorization-based unified framework

Similar to DDINMF, Shi *et al*. [99] presented a triple matrix factorization-based unified framework (TMFUF) to infer both enhancive and degressive DDIs. For *m* known drugs, the adjacency matrix *A* was constructed in the same way as in DDINMF, but in TMFUF, the feature vectors were constructed only based on the side effect information of drugs. Then, the author modeled the relationship between matrix *A* and feature matrix *F* as a bi-linear regression, which could be represented as the triple matrix factorization:


(22) \begin{equation*} A= F\varTheta{F}^T \end{equation*}

where $\varTheta$ refers to the symmetrical projection matrix, whose role is to link the features of drugs with the interactions between drugs. To obtain the matrix $\varTheta$, the author first calculated matrix ${A}_d^{\ast }$ as follows:


(23) \begin{equation*} {A}_d^{\ast }=\underset{A_d}{\arg \min }{\left\Vert A-{A}_d{A_d}^T\right\Vert}^2 \end{equation*}

where *A_d_* denotes the latent interaction matrix, whose row refers to the feature of corresponding drug in the latent space, and singular value decomposition was used to obtained ${A}_d^{\ast }$. Then, matrix ${B}^{\ast }$ was calculated:


(24) \begin{equation*} {B}^{\ast }=\underset{B}{\arg \min }{\left\Vert{A}_d^{\ast }- FB\right\Vert}^2 \end{equation*}

where *B* represents the regression coefficient matrix. SIMPLS [98] was applied to solve the optimization problem to obtain ${B}^{\ast }$, and the matrix $\varTheta$ was obtained as follows:


(25) \begin{equation*} \varTheta ={B}^{\ast }{B}^{\ast^T} \end{equation*}

Then, the interaction possibility matrixes could be calculated:


(26) \begin{equation*} {P}_{n,n}={F}_n\varTheta{F_n}^T \end{equation*}

(27) \begin{equation*} {P}_{n,m}={F}_n\varTheta{F}^T \end{equation*}

where *F_n_* represents the feature matrix involving only new drugs; the elements of *P_n,n_* represent the interaction probabilities between new drugs, while the elements of *P_n,m_* refer to the interaction probabilities between new drugs and known drugs. That is, TMFUF could be used to predict not only known but also new drugs that interact with new drugs.

#### Local classification model via Dempster–Shafer theory of evidence

Under the assumption that similar drugs tend to interact with the same drug, Shi *et al*. [100] also proposed an integrated local classification model via Dempster–Shafer theory of evidence (LCM-DS) to predict potential DDIs. The authors first constructed the drug similarity matrix through directly averaging three different drug similarity matrices (i.e. chemical structures-based, side effect-based and off-label side effect-based drug similarity matrix) derived from the work of Zhang *et al*. [101]. Then, based on the known DDIs as well as drug similarity, three local classification-based models (LCMs) constructed according to SVM [102], regularized least squares (RLS) [103] and multi-label K-nearest neighbors (MLKNNs) [104], were applied to calculate the interaction probabilities between drugs, respectively. Finally, the authors proposed a novel fusion method based on the Dempster–Shafer theory of evidence [105] to integrate the results of the three LCMs to obtain the final interaction probabilities between drugs.

#### D‌DIGIP

Based on the Gaussian Interaction Profile (GIP) kernel and RLS classifier, Yan *et al*. [106] proposed a model of DDIGIP to predict potential DDIs. Specifically, the authors first calculated eight types of drug feature vectors in the same way as MRMF, which were spliced to form the final feature vectors of drugs. Then, they used the Pearson correlation coefficient to calculate the similarities between drugs based on their respective feature vectors and then constructed the drug similarity matrix *S_P_*. Later, to make DDIGIP also applicable to new drugs, the authors used the K-nearest neighbors (KNNs) to calculate the initial relational score between new drug *d_i_* and known drug *d_j_* to fill in the adjacency matrix *A*:


(28) \begin{equation*} A\left({d}_i,{d}_j\right)=\frac{\sum{S}_P\left({d}_i,{d}_l\right)A\left({d}_l,{d}_j\right)}{\sum{S}_P\left({d}_i,{d}_l\right)},{d}_l\in{K}_{set}^i \end{equation*}

where ${K}_{set}^i$ represents the set of top *K* drugs with the largest similarity to drug *d_i_*. Next, they calculated the drug GIP similarity matrix *S_G_* via the filled adjacency matrix. Finally, the authors used the RLS classifier to compute the predicted interaction probability matrix *P* as follows:


(29) \begin{equation*} {P}^{\prime}={S}_G{\left({S}_G+\sigma I\right)}^{-1}A \end{equation*}

(30) \begin{equation*} P=\frac{P^{\prime}+P{\hbox{'}}^T}{2} \end{equation*}

where *I* refers to the identity matrix and $\sigma$ represents the regularization parameters.

#### Gradient boosting-based model

**Figure 3:**
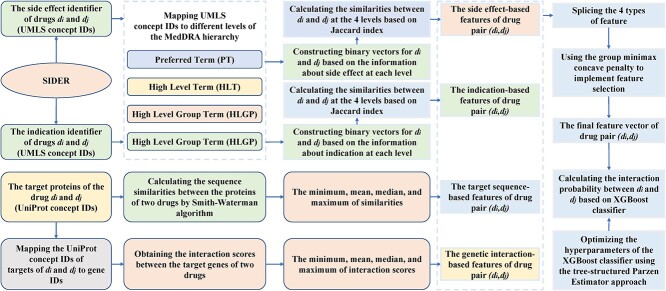
The flowchart of the gradient boosting-based model, where XGBoost classifier is applied to predict potential DDIs based on multiple features of drug pairs.

Qian *et al*. [107] proposed an extreme gradient boosting (XGBoost) classifier for the prediction of DDIs by integrating multiple features of drug pairs (Figure 3). Different from the way of constructing the feature vectors of drugs directly based on the side effect information in the previous models, the authors downloaded the data on side effects from SIDER [66], where the Unified Medical Language System (UMLS) concept IDs [108] were used as the side effects identifiers. Then, according to the dictionary MedDRA [109], they mapped the UMLS concept IDs to MedDRA concept IDs at four different levels, including preferred term (PT), high-level term (HLT), high-level group term (HLGP) and system organ class (SOC). At each level, a binary vector was constructed for each drug as its feature vector. Furthermore, the Jaccard index was used to calculate the similarities between two drugs at the four levels, respectively, which constituted the side effect-based features of corresponding drug pairs. The information about drug indications was also collected from SIDER [66] and mapped to the same four levels. Then, the indication-based features of drug pairs were obtained in a similar way. Moreover, the authors calculated the sequence similarities between the target proteins of two drugs by Smith–Waterman algorithm [110] and then used the minimum, mean, median and maximum of the similarities between target proteins to construct the target sequence-based features of corresponding drug pairs. Similarly, the interaction scores between genes were downloaded from the study of Costanzo *et al.* [111], and the minimum, mean, median as well as maximum of interaction scores between the target protein gene of two drugs were used to construct genetic interaction-based features of the corresponding drug pairs. Therefore, 16 features of each drug pair could be obtained by integrating the above four types of features. Then, a feature selection method known as group minimax concave penalty (MCP) [112] was applied to obtain 11 features with significantly different value distributions between interacting drug pairs and noninteracting drug pairs, which formed the final feature vector for each drug pair. Finally, the authors applied the XGBoost classifier to calculate the interaction probability between corresponding drugs. In addition, to obtain better prediction performance, the authors optimized the hyperparameters of the classifier using the tree-structured Parzen estimator (TPE) approach [113].

#### Network algorithm and matrix perturbation algorithm-based model

By means of multisource data fusion, Zhang *et al.* [114] presented a flexible framework to integrate multiple models for DDI identification. Firstly, the authors applied the Jaccard index to calculate eight types of drug similarities based on the information about substructure, target, enzyme, transporter, pathway, indication, side effect and off side effect of drugs, respectively. Besides, according to the DDIs network constructed based on known DDIs, they computed six other kinds of drug similarities, namely, common neighbor similarity, Adamic–Adar similarity, resource allocation similarity, Katz similarity, average commute time similarity and random walk with restart similarity. Furthermore, the authors adopted two similarity-based models (constructed based on the neighbor recommender algorithm [115] as well as the random walk algorithm [116]) and one model only based on known DDIs (built according to the matrix perturbation algorithm [117]) to predict potential DDIs. Thus, according to the above-mentioned 14 types of drug similarities and known DDIs, 29 prediction models, namely, 28 similarity-based models and one model, based only on known DDIs were constructed. Finally, the weighted average ensemble rule and the classifier ensemble rule were implemented to fuse the prediction results, respectively. Specifically, the weighted average ensemble rule took the weighted average of the outputs of all prediction models, while the classifier ensemble rule took the logistic regression to map the outputs of all models to a score as the final prediction results.

#### Heterogeneous network-assisted inference

Cheng *et al*. [118] proposed a heterogeneous network–assisted inference (HNAI) framework to identify potential DDIs. The authors firstly extracted 6 946 known DDIs from DrugBank [6] as positive samples, which formed the training set with the same number of drug pairs randomly selected from drug pairs without verified interaction. Then, the authors calculated four types of drug similarities: phenotypic similarity, therapeutic similarity, chemical structural similarity and genomic similarity. Specifically, the phenotypic similarity, therapeutic similarity and chemical structural similarity were calculated according to the method proposed in the authors’ previous work [119], respectively. As for genomic similarity, the authors firstly constructed a binary vector for each drug based on the target protein information, and then the Tanimoto coefficient [120] of the two binary vectors was regarded as the genomic similarity between the corresponding two drugs. The four types of similarity between the two drugs constituted the feature vector of the corresponding drug pair. Finally, based on the drug pairs in the training set and corresponding feature vectors, the authors trained five prediction models (namely, naive Bayes, decision tree, KNN, logistic regression and SVM) to identify potential DDIs, respectively. Moreover, the authors constructed the interaction network based on the known DDIs and carried out statistical analysis by combining the above four types of similarity. It turned out that the more similar two drugs are, the higher probability of interaction between them.

#### Integrated action crossing

Hunta *et al*. [121] proposed an integrated action crossing (IAC) method to predict the potential DDIs by focusing on the drug–enzyme and drug–transporter actions. Different from the above models where the feature vectors of drug pairs were constructed based on drug similarity, the authors proposed a method called action crossing (AC) to obtain the feature vectors of drug pairs according to the information about drug-enzyme actions [including substrate (S), inhibitor (Inh) as well as inducer (Inc)]. Specifically, for drug *d_i_* and enzyme *e_k_*, the action attribute vector *X^ik^* was defined as follows:


(31) \begin{equation*} {X}^{ik}=\left[{x}_S^{ik},{x}_{Inh}^{ik},{x}_{Inc}^{ik}\right] \end{equation*}

where ${x}_S^{ik}$,${x}_{Inh}^{ik}$ and ${x}_{Inc}^{ik}$ represent whether the drug *d_i_* has a corresponding action on the enzyme *e_k_*. Taking ${x}_S^{ik}$ for an example, if drug *d_i_* is a substrate of enzyme *e_k_*, the value of ${x}_S^{ik}$ is 1, otherwise 0. ${x}_{Inh}^{ik}$ and ${x}_{Inc}^{ik}$could be obtained in a similar way. Then, the feature vector of drug pair (*d_i_,d_j_*) based on enzyme *e_k_* was constructed:


(32) \begin{equation*} {F}_{ij}^k=\left[{f}_1^{ij k},{f}_2^{ij k},{f}_3^{ij k}\right] \end{equation*}

where if the *p*th (*p* = 1–3) element of vectors *X^ik^* and *X^jk^* were both 1, ${f}_p^{ijk}$ has a value of 1, otherwise 0. Similarly, they constructed transport-based feature vectors of drug pairs based on the information about drug–transporter actions. The authors collected 36 enzymes as well as 35 transporters and sorted them based on their ID. Then, they calculated the feature vector based on enzyme and transporter in turn and spliced them to obtain the final feature vector. Finally, the final feature vectors of drug pairs were used to train three models (SVM, KNN and neural networks), respectively, which were used to predict potential DDIs.

#### SFLLN

Zhang *et al.* [122] proposed the sparse feature learning ensemble method with linear neighborhood regularization, named SFLLN, to predict potential DDIs. Firstly, the authors constructed the corresponding binary feature vectors for each drug based on the information about substructure, target, enzyme and pathway, respectively. Then, the same type of feature vectors for all drugs were combined into the feature matrix *F_i_* (*i* = 1–4). Secondly, the authors projected drugs from different feature spaces to the common interaction space by approximating the interaction probability matrix *P* with the product of the feature matrix *F_i_* and the nonnegative projection matrix *G_i_*. Besides, they controlled the sparsity of projection matrixes through minimizing $\sum \limits_{k=1}^{n_i}{\left\Vert{G}_i\left[,k\right]\right\Vert}^2$to improve the generalization ability of the model, where *n_i_* referred to the number of columns in matrix *G_i_*. Therefore, they defined the objective function as follows:


(33) \begin{equation*} \underset{G,P}{\min }{\left\Vert A-P\right\Vert}_F^2+\mu \sum \limits_{i=1}^4{\left\Vert{F}_i{G}_i-P\right\Vert}_F^2+\lambda \sum \limits_{i=1}^4\sum \limits_{k=1}^{n_i}{\left\Vert{G}_i\left[,k\right]\right\Vert}^2 \end{equation*}

where *A* refers to the adjacency matrix constructed based on known DDIs; $\lambda$ and $\mu$ refer to free parameters. Besides, by assuming that the predicted DDIs have the same structure as the known DDIs, the authors defined the Lagrangian function as follows to extract the data structure from known DDIs:


(34) \begin{equation*} L=\frac{1}{2}{\left\Vert A-\left(C\otimes W\right)A\right\Vert}_F^2+\frac{\mu }{2}{\left\Vert \left(C\otimes W\right)e\right\Vert}_2^2-\lambda \left(\left(C\otimes W\right)e-e\right)- tr\left(\varPhi W\right) \end{equation*}

where $\otimes$ denotes the Hadamard product; *C* is an indicator matrix, where *C*[*i,j*] = 0 if *i = j*, otherwise *C*[*i,j*] = 1; *e* is a *n_d_*-dimensional vector with all elements being 1; *n_d_* is the total number of drugs; $\varPhi$ refers to the Lagrange multiplier; matrix *W* reflects the intrinsic structure of known DDIs. Then, *W* was obtained by taking the derivative of *L* with respect to *W* and setting the derivative to 0. In order to ensure that the drugs retain their internal structure after projection, matrixes *P* and *W* should meet the following requirement:


(35) \begin{equation*} P\left[i,j\right]-\sum \limits_{k=1}^{n_d}W\left[i,k\right]P\left[k,j\right]\approx 0 \end{equation*}

Therefore, by algebraic transformation, the authors defined the linear neighborhood regularization [123]:


(36) \begin{equation*} \sum \limits_{i=1}^{n_d}\sum \limits_{j=1}^{n_d}P\left[i,j\right]\left(P\left[i,j\right]-\sum \limits_{k=1}^{n_d}W\left[i,k\right]P\left[k,j\right]\right)= tr\left({P}^T\left(I-W\right)P\right) \end{equation*}

By combining Formulas (33) and (36), the authors defined the final objective function:


(37) \begin{equation*} \underset{G,P}{\min }{\left\Vert A-P\right\Vert}_F^2+\mu \sum \limits_{i=1}^4{\left\Vert{F}_i{G}_i-P\right\Vert}_F^2+\delta tr\left({P}^T\left(I-W\right)P\right)+\lambda \sum \limits_{i=1}^4\sum \limits_{k=1}^{n_d}{\left\Vert{G}_i\left[,k\right]\right\Vert}_1^2 \end{equation*}

The authors set the partial derivative of *L* to *P* as 0 to obtain the relationship between *P* and *G*:


(38) \begin{equation*} P={\left(\delta \left(I-W\right)+\left(1+ m\mu \right)I\right)}^{-1}\left(A+\mu \sum \limits_{k=1}^4{F}_k{G}_k\right) \end{equation*}

Then, Formula (37) could be rewritten as follows:


(39) \begin{equation*} {\displaystyle \begin{array}{l}\underset{G}{\min}\sum \limits_{k=1}^4 tr\Big(\mu \left({G}_k^T{F}_k^T{F}_k{G}_k-2\mu{A}^T{\left(\delta \left(I-W\right)+\left(1+ m\mu \right)I\right)}^{-1}{F}_k{G}_k+\lambda{G}_k^T{E}_k{G}_k\right)\\{}-{\mu}^2\sum \limits_{k_1=1}^4\sum \limits_{k_2=1}^4 tr\left({G}_{k_1}^T{F}_{k_1}^T{\left({\left(\delta \left(I-W\right)+\left(1+ m\mu \right)I\right)}^{-1}\right)}^T{F}_{k_2}{G}_{k_2}\right)\end{array}} \end{equation*}

where all elements in matrix ${E}_k\in{R}^{n_k\times{n}_k}$ are equal to 1 and *n_k_* represents the number of columns in *F_k_*. Finally, the authors solved Formula (39) by semi-nonnegative matrix factorization algorithm to get *G_k_* and then obtained the interaction probability matrix *P* based on Formula (38).

### Deep learning-based models

Given the successful application of deep learning algorithms in the fields such as natural language processing (NLP) [124, 125] and pattern recognition [126, 127] in recent years, more and more researchers have built models based on deep learning methods to predict potential DDIs. Deep learning-based models can not only automatically extract the features of drugs but also effectively integrate the features of drugs by multiple modules to obtain the features of the corresponding drug pairs. In addition, NLP-based models could be applied to mine a large number of DDIs from the literatures. However, as with the models constructed based on the traditional machine learning algorithms, the scarcity of reliable negative samples severely limits the performance of deep learning-based models. Besides, because the goal of training is to obtain the optimal values of the parameters and the most significant features of the drug pairs, using deep learning-based models usually takes more time to make predictions. We have a brief introduction to some of them below.

#### D‌DIMDL

Deng *et al.* [128] developed a multimodal deep learning framework to identify unknown DDIs (DDIMDL). Firstly, the authors constructed the corresponding binary feature vector for each drug based on the information about substructure, target, enzyme and pathway, respectively. Secondly, the Jaccard index was used to calculate the similarities between drugs and four corresponding similarity matrices (i.e. *S_s_*, *S_t_*, *S_e_* and *S_p_*) were constructed later, where the *i*th row of each similarity matrix was regarded as the corresponding feature vector of drug *d_i_*. Thirdly, for each similarity matrix, its *i*th and *j*th rows were fed into the DNN to calculate the interaction probability between *d_i_* and *d_j_*. Finally, the authors took the average of the interaction probabilities obtained based on the four similarity matrices as the final result.

#### Substructure–substructure interaction for drug–drug interaction

**Figure 4:**
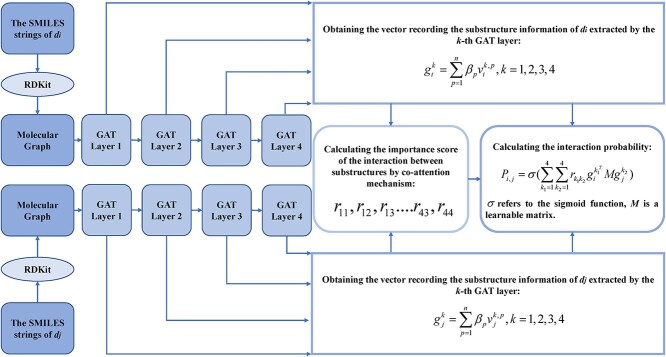
The flowchart of SSI-DDI, which is applied to predict potential DDIs based on the interactions between drug substructures.

Considering that DDIs were caused by the interactions between the substructures of the corresponding two drugs, Shi *et al.* [129] developed a deep learning-based model named substructure–substructure interaction for drug–drug interaction (SSI-DDI) to predict potential DDIs (Figure 4). Firstly, for drug *d_i_*, based on the software RDKit (https://www.rdkit.org/), the SMILES string of *d_i_* download from DrugBank [6] was converted into a molecular graph, where nodes represented the atoms contained in *d_i_* and edges referred to the bonds between corresponding atoms. Secondly, they built a module made up of four graph attention (GAT) layers in series (layer 1$\to$layer 2$\to$ layer 3$\to$layer 4), where layer 1 was used to extract the feature vector (64-dimension) of each node based on the molecular graph, while the other three layers were applied to update the vector output from the previous layer. Then, by integrating the feature vectors of all atom nodes in the molecular graph of *d_i_*, the vector recording the substructure information extracted by the *k*th layer could be obtained:


(40) \begin{equation*} {g}_i^k=\sum \limits_{p=1}^n{\beta}_p{v}_i^{k,p} \end{equation*}

where *n* is the total number of nodes; parameter ${\beta}_p$ indicates the importance of the *p*th node, which could be obtained by SAGPooling [130]; ${v}_i^{k,p}$ refers to the feature vector of the *p*th node extracted by the *k*th layer. For the substructure of *d_i_* extracted by the *k_1_*th layer and substructure of *d_j_* extracted by the *k_2_*th layer, the co-attention mechanism was applied to calculate the importance score ${r}_{k_1{k}_2}$of the interaction between these two substructures to the final DDI prediction. Finally, the interaction probability between *d_i_* and *d_j_* could be obtained by integrating the interactions between substructures of two drugs:


(41) \begin{equation*} {P}_{i,j}=\sigma \left(\sum \limits_{k_1=1}^4\sum \limits_{k_2=1}^4{r}_{k_1{k}_2}{g}_i^{{k_1}^T}M{g}_j^{k_2}\right) \end{equation*}

where $\sigma$ refers to the sigmoid function; *M* is a learnable matrix, which was obtained by training SSI-DDI on 1024 known DDIs using the Adam optimizer [131].

#### Substructure-aware tensor neural network model for DDI prediction

Shi *et al.* [132] also constructed another model named substructure-aware tensor neural network model for DDI prediction (STNN-DDI), which could be used to predict the types of DDI. Firstly, each drug was represented by an *n*-dimensional binary vector, where *n* represents the total number of substructures under study. For example, drug *d_i_* could be denoted by the vector ${e}_i=\left[{e}_1^i,{e}_2^i,...{e}_n^i\right]$, where the value of ${e}_j^i$ is 1 if *d_i_* contains the *j*th substructure and 0 otherwise. Then, for drugs *d_i_* and *d_j_*, the authors used ${P}_{ijk}^d$ to represent the probability of the *k*th type of interaction between *d_i_* and *d_j_*, which could be defined as follows:


(42) \begin{equation*} {P}_{ijk}^d=\sum \limits_{p=1}^n\sum \limits_{q=1}^n{P}_{pqk}^s{e}_p^i{e}_q^j \end{equation*}

where ${P}_{pqk}^s$ refers to the probability that there is *k*th type of interaction between the *p*th substructure and the *q*th substructure. In order to get the interaction probabilities between substructures, the authors constructed a tensor named *ST*, where both axes *x* and *y* represented the substructures, while the axis *z* referred to the DDI types, and set $S{T}_{pqk}={P}_{pqk}^s$. Therefore, Formula (42) could be rewritten as


(43) \begin{equation*} {P}_{ijk}^d=\sum \limits_{p=1}^n\sum \limits_{q=1}^nS{T}_{pqk}{e}_p^i{e}_q^j \end{equation*}

Based on the CP decomposition [133], the tensor *ST* could be approximated by three factor matrices *A, B* and *C*:


(44) \begin{equation*} ST\approx \sum \limits_{z=1}^r{\lambda}_z\left({a}_z\times{b}_z\times{c}_z\right)=\left\langle \lambda; A,B,C\right\rangle \end{equation*}

where $\lambda$ denotes the *r*-dimensional vector used to normalize the columns of three factor matrices; $A\in{R}^{n\times r}$,$B\in{R}^{n\times r}$ and $C\in{R}^{f\times r}$record the latent information of the *x*, *y* and *z* axes, respectively; *r* is the number of rank-one tensors decomposed by *ST*; *f* represents the total number of DDI types. Besides, by assuming that the rows of matrix *A* and *B* represent the embedding of the corresponding substructure, while the rows of *C* refer to the embedding of the corresponding DDI type, the authors introduced the multi-linear tensor transformation [134] to define the interaction probability ${P}_{ijk}^{d^{\ast }}$ of the *k*th type of interaction between *d_i_* and *d_j_*:


(45) \begin{equation*} {\displaystyle \begin{array}{l}{P}_{ijk}^{d^{\ast }}=\sum \limits_{z=1}^r{\lambda}_z\left({a}_z\times{b}_z\times{c}_z\right){\overline{\times}}_3{v}_k{\overline{\times}}_2{e}_i{\overline{\times}}_1{e}_j+ bias\\{}\kern1.5em =\left[\left({e}_i^T\times A\right)\otimes \left({e}_j^T\times B\right)\otimes \left({v}_k^T\times C\right)\right]\lambda + bias\end{array}} \end{equation*}

where ${\overline{\times}}_n$ refers to the mode-*n* product; *v_k_* represents the one-hot vector encoding the *k*th type of interaction; parameter *bias* is added to enhance the robustness of STNN-DDI. Then, they defined the loss function *F*:


(46) \begin{equation*} F=\sum \limits_{\left({d}_i,{d}_j\right)\in{D}_{train}}{\left(P{D}_{ijk}-{P}_{ijk}^{d^{\ast }}\right)}^2 \end{equation*}

where *D_train_* is the training set consisting of all positive samples and an equal number of randomly selected negative samples; the value of *PD_ijk_* is 1 if there is the *k*th type of interaction between drug *d_i_* and *d_j_* and 0 otherwise. Finally, the authors constructed a fully connected neural network model based on Formulas (45) and (46) to obtain matrices *A, B, C*, $\lambda$ and *bias* according to the training set. Then, the interaction probability between drugs could be calculated based on Formula (45).

#### META-DDIE

Deng *et al.* [135] proposed a few-shot computational model named META-DDIE to predict the types of DDIs, which consisted of a representation module and a comparing module. In the representation module, the authors first constructed a binary vector *S_i_* for drug *d_i_* based on its structure information. For drug pair *d_i_–d_j_*, its feature vector *F_i,j_* was defined as follows:


(47) \begin{equation*} {F}_{i,j}\left[k\right]=\left\{\begin{array}{c}0, {\rm if}\ {S}_i\left[k\right]=0\ {\rm and}\ {S}_j\left[k\right]=0\\{}1, {\rm otherwise} \end{array}\right. \end{equation*}

Then, the authors employed a neural network to encode the vector *F_i,j_* to a embedding vector ${E}_{i,j}^1$ and applied another neural network to decode a new feature vector ${F}_{i,j}^{\prime}$ from vector ${E}_{i,j}^1$. To train the framework consisting of encoder and decoder, the authors defined the loss function:


(48) \begin{equation*} {L}_{i,j}=\sum \limits_{k=1}^n\left({F}_{i,j}\left[k\right]\log \left({F}_{i,j}^{\prime}\left[k\right]\right)+\left(1-{F}_{i,j}\left[k\right]\right)\log \left(1-{F}_{i,j}^{\prime}\left[k\right]\right)\right) \end{equation*}

where *n* is the dimension of the vector *F_i,j_*. Secondly, based on the SMILES of drugs, the authors applied a chemical sequential pattern mining (SPM) algorithm [136] to obtain a set of discrete frequent substructures of drugs in the database. The *k*th frequent substructure (denoted by a single-hot vector *v_k_*) was fed into the above neural network for encoding to obtain corresponding embedding vector ${E}_k^2$. Then, the vector ${E}_{i,j}^1$ could be projected on a subspace defined by span ($\left[{E}_1^2,{E}_2^2,...,{E}_{n_s}^2\right]$):


(49) \begin{equation*} {E}_{i,j}^1={E}_1^2{r}_{i,j}^1+{E}_2^2{r}_{i,j}^2+...+{E}_{n_s}^2{r}_{i,j}^{n_s} \end{equation*}

where ${r}_{i,j}^k$ (*k* = 1,2,…,*n_s_*) represents the projection coefficient, which could be calculated via the method proposed by Huang *et al.* [137]; *n_s_* denotes the total number of frequent substructures. The vector ${r}_{i,j}=\left[{r}_{i,j}^1,{r}_{i,j}^2,...,{r}_{i,j}^{n_s}\right]$ was regarded as the final representation the drug pair *d_i_–d_j_*. For the few-shot learning, the authors divided drug pairs into training sets and test sets, and both of them were further divided into support set as well as query set. For drug pairs *DP_p_* in the support set and *DP_q_* in the query set, their representation were fed into the comparing module constructed as in the study [138] to obtain an *n_t_*-dimensional similarity vector *S_p,q_* between the two drug pairs, where *n_t_* refers to the number of DDI types. Then, they defined the loss function based on mean square error to train the model:


(50) \begin{equation*} L=\sum \limits_{p=1}^{n_p}\sum \limits_{q=1}^{n_q}{\left(\max \left({S}_{p,q}\right)-{l}_{p,q}\right)}^2 \end{equation*}

where *n_p_* and *n_q_* represent the number of drug pairs in the support set and the query set, respectively. If *DP_p_* and *DP_q_* have the same type of DDIs, the value of *l_p,q_* is 1, otherwise 0. Then, the model was trained by minimizing the loss function *L*. Finally, the authors applied the trained model to calculate the similarity vector *S_x,y_* between drug pairs *DP_x_* in the support set and *DP_y_* in the query set. The DDIs type corresponding to the maximum value in the vector *S_x,y_* was regarded as the type of drug pair *DP*_*y*._

#### Deep attention neural network-based drug–drug interaction prediction model

Liu *et al.* [139] developed a deep attention neural network-based drug–drug interaction prediction model (DANN-DDI) to identify potential DDIs. Specifically, the authors first constructed five networks, including the drug–substructure network, drug–target network, drug–enzyme network, drug–pathway network and DDI network. For drug *d_i_*, the authors applied structural deep network embedding method [140, 141] to learn its embeddings (i.e. ${E}_i^s$,${E}_i^t$,${E}_i^e$,${E}_i^p$ and${E}_i^d$) from the above networks, respectively. Then, the authors constructed the comprehensive vector ${E}_i=\left[{E}_i^s,{E}_i^t,{E}_i^e,{E}_i^p,{E}_i^d\right]$ of *d_i_* based on the above embeddings. For drug *d_i_* and *d_j_*, their comprehensive vectors were used as the input of the attention neural network [142] to obtain the feature vector of drug pair *d_i_–d_j_*. Finally, the feature vector was fed into the framework consisting of the input layer, multiple fully connected hidden layers and the output layer, and the softmax function was applied to calculate the interaction probability between *d_i_* and *d_j_* based on the output of the framework.

#### Multi-relational contrastive learning graph neural network

Xiong *et al.* [143] proposed a model named multi-relational contrastive learning graph neural network (MRCGNN) to predict the types of DDIs. Firstly, by taking drugs as nodes and known DDIs as edges, the authors constructed a multi-relational DDI event graph *G = (V,E,T)*, where *V* and *E* represent the set of all drug nodes and edges, respectively, *T* denotes the set of all DDIs types. Secondly, after obtaining the molecular graph of each drug in the same way as used in SSI-DDI, the authors utilized TrimNet [144] to extract the features of drugs based on the corresponding molecular graph and constructed feature matrix *F* by combining the features of all drugs*.* Then, the authors employed the relational graph convolutional network (R-GCN) encoder [145] to learn the original representation vectors of drugs from the graph *G* with the features of drugs as node attributes, and the representations of all drugs formed the matrix *H*. Besides, the global representation *g* was defined:


(51) \begin{equation*} g=\varGamma (H) \end{equation*}

where Γ refers to the readout function. Thirdly, in order to implement the multi-relational contrastive learning on *G*, the authors corrupted the graph *G* by shuffling the features of drug nodes and edges to obtain corrupted graphs ${G}_v=\left(\tilde{V},E,T\right)$ and ${G}_e=\left(V,\tilde{E},T\right)$, which were fed into the R-GCN encoder to obtain the corresponding drug representation matrix *H_v_* and *H_e_*, respectively. Given that the training goal of contrastive learning was to maximize the consistency between *H* and *g*, as well as the difference between *H_v_/H_e_* and *g*, the authors defined two loss functions:


(52) \begin{align*} {L}_v=&-\frac{1}{\left|V\right|+\left|\tilde{V}\right|}\left(\sum \limits_{i=1}^{n_d}{E}_{\left(V,E,T\right)}\left[\log D\left(H\left[i,\right],g\right)\right]\right.\nonumber\\&\left.+\sum \limits_{i=1}^{n_d}{E}_{\left(\tilde{V},E,T\right)}\left[\log \left(1-D\left({H}_v\left[i,\right],g\right)\right)\right]\right) \end{align*}

(53) \begin{align*} {L}_e=&-\frac{1}{\left|V\right|+\left|V\right|}\left(\sum \limits_{i=1}^{n_d}{E}_{\left(V,E,T\right)}\left[\log D\left(H\left[i,\right],g\right)\right]\right.\nonumber\\ &\left.+\sum \limits_{i=1}^{n_d}{E}_{\left(V,\tilde{E},T\right)}\left[\log \left(1-D\left({H}_e\left[i,\right],g\right)\right)\right]\right) \end{align*}

where $D\left(H\left[i,\right],g\right)=\sigma H{\left[i,\right]}^T Wg$; *W* represents a trainable parameter matrix; *n_d_* refers to the total number of drugs. For drug pair *d_i_–d_j_*, the authors spliced the corresponding features and representations (i.e. *F*[*i*,], *F*[*j*,], *H*[*i*,] and *H*[*j*,]) together to obtain the final representations *r_i,j_* of the drug pair, which was fed into the multilayer perceptron (MLP) followed by a Softmax function to implement multi-class prediction:


(54) \begin{equation*} {P}_{i,j}= soft\max \left( MLP\left({r}_{i,j}\right)\right) \end{equation*}

where *P_i,j_* is a *n_t_*-dimensional vector; *n_t_* is the number of DDIs types; *P_i,j_*[*k*] represents the probability of the *k*th type of interaction between drug *d_i_* and *d_j_*. Then, the authors defined another loss function:


(55) \begin{equation*} {L}_c=-\sum \limits_{\left({d}_i,{d}_j\right)\in \varOmega}\sum \limits_{k=1}^{n_t}{L}_{i,j}^k\log{P}_{i,j}\left[k\right] \end{equation*}

where $\varOmega$ represents the training set; ${L}_{i,j}^k$ is the true label of drug pair *d_i_–d_j_*, if there is the *k*th type of interaction between *d_i_* and *d_j_*, ${L}_{i,j}^k$ has the value 1, otherwise 0. To train MRCGNN, the authors defined the final loss function based on the above three loss functions:


(56) \begin{equation*} L={L}_c+\alpha{L}_v+\beta{L}_e \end{equation*}

where $\alpha$ and $\beta$ refer to the hyperparameters used to balance different loss functions.

#### Multichannel feature fusion model for multi-typed DDI prediction

**Figure 5:**
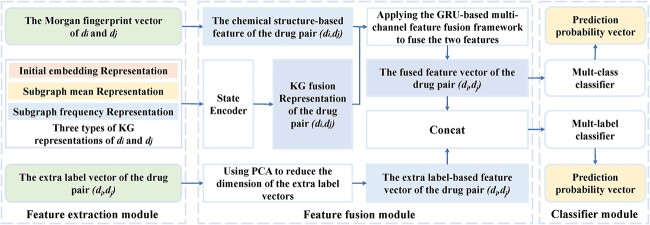
The flowchart of MCFF-MTDDI, which consists of three modules: feature extraction module, feature fusion module and classifier module.

Chen *et al.* [146] developed a multichannel feature fusion model for multi-typed DDI prediction (MCFF-MTDDI), which consisted of three modules, namely, feature extraction module, feature fusion module and classifier module (Figure 5). The authors firstly removed all <DRUGBANK::ddi-interactor-in::Compound::Compound> edges to remove the DDI information from the drug repurposing knowledge graph (DRKG) [147]. The remaining triples made up the biomedical knowledge graph (KG) dataset after removing the isolated drug nodes. In the feature extraction module, the authors extracted three types of KG representations (namely, initial embedding representation, subgraph mean representation and subgraph frequency representation) for each drug through corresponding methods based on the KG dataset, respectively. Besides, they also obtained the Morgan fingerprint vector [148] of each drug through RDKit (https://www.rdkit.org/) based on the SMILES string of corresponding drug. Then, the Morgan fingerprint vectors of the two drugs were spliced together to construct the chemical structure-based feature of the corresponding drug pair. Moreover, the authors constructed the extra label-based feature of each drug pair for multi-label prediction. Specifically, after removing the drugs without SMILES, there were 12 362 different DDI types in the dataset, corresponding to 12 362 labels. Then, from the DDI labels involving more than 10 000 drug pairs, the authors selected 200 labels involving the fewest drug pairs to build the target label set, and the remaining 12 162 labels made up the extra label set. For the drug pair *(d_i_,d_j_)*, a 12 162D binary vector ${H}_{EL\left({d}_i,{d}_j\right)}=\left[{h}_{ij}^1,{h}_{ij}^2,...,{h}_{ij}^{12162}\right]$ was constructed as its extra label vector, where if there was *k*th type of interaction between *d_i_* and *d_j_*, the value of ${h}_{ij}^k$ is 1, otherwise 0. Then, principal component analysis (PCA) was applied to reduce the dimension of extra label vector to obtain vector ${H}_{EL\left({d}_i,{d}_j\right)}^{\prime}\in{R}^{300}$, and the extra label-based feature vector was defined:


(57) \begin{equation*} {F}_{EL\left({d}_i,{d}_j\right)}=\operatorname{Re} LU\left(W{H}_{EL\left({d}_i,{d}_j\right)}^{\prime}+b\right) \end{equation*}

where *W* represents the trainable weight and *b* refers to the bias. In the feature fusion module, the state encoder consisting of two fully connected layers and two state vector strategy blocks was used to integrate the KG representations to obtain the KG fusion representations of the drug pair (*d_i_,d_j_*). Moreover, the chemical structure-based feature and KG fusion representations were input into a GRU-based multichannel feature fusion framework to obtain the fused feature vector ${F}_{FU\left({d}_i,{d}_j\right)}$ of the drug pair (*d_i_,d_j_*). In the classifier module, the vector ${F}_{FU\left({d}_i,{d}_j\right)}$ was used as the input of the multi-class classifier to implement the multi-class classification tasks, while the extra label-based feature vector ${F}_{EL\left({d}_i,{d}_j\right)}$ and vector ${F}_{Fu\left({d}_i,{d}_j\right)}$ were concatenated and input into the multi-label classifier to implement the multi-label classification. It should be pointed out that both classifiers consisted of two fully connected layers, where the number of neurons in the last layer was equal to the number of DDI types of the corresponding classification task.

#### DSIL-DDI

Tang *et al.* [149] proposed a model called DSIL-DDI to predict potential DDIs by implementing causal representation learning [150] on the substructures of drugs. Specifically, for drug *d_i_*, the authors first constructed the molecular graph for *d_i_* in the same way as used in SSI-DDI, which was input into graph neural networks (GNNs) to obtain its substructure representations, where the *p*th substructure was represented by vector ${S}_i^p$. After obtaining the representations of two substructures, the priori representation of the interaction between them was defined:


(58) \begin{equation*} ss{i}_{pq}={W}_{SSI}{S}_i^p\otimes{S}_j^q \end{equation*}

where *W_SSI_* represents the learnable weight matrix. Then, the attention weights were used to modify the priori representation to obtain the posteriori representation:


(59) \begin{equation*} postss{i}_{pq}= MLP\left( concat\left({S}_i^p,{S}_j^q\right)\right)\times ss{i}_{pq} \end{equation*}

where *MLP* denotes the multilayer perceptron and *concat* refers to the concatenate operation. In a similar way, the authors calculated the posteriori representations between all substructures of *d_i_* and all substructures of *d_j_*, which were integrated to obtain the substructure interaction matrix, where the row and column represented the substructure of two drugs, respectively, and the element referred to the posteriori representation of corresponding two substructures. Finally, the substructure interaction matrix was input into the single-layer linear network to obtain the interaction probability between *d_i_* and *d_j_*.

#### DSN-DDI

Based on the intra-view and inter-view representation learning methods, Li *et al.* [151] developed a novel model named DSN-DDI to identify potential DDIs. Firstly, the authors obtained the molecule graph of each drug in the same way as used in SSI-DDI. Besides, for the drug pair (*d_i_,d_j_*), they built a bipartite graph by connecting each atom node in the molecule graph of *d_i_* with all nodes in the molecule graph of *d_j_* in turn. Secondly, to learn the representations of nodes in the graphs, the authors constructed four identical DSN encoders (composed of the representation extraction layer, the intra-view layer and the inter-view layer), which formed the DNS encoder module in series. Specifically, in the first DSN encoder, the molecular graph of drug *d_i_* was input into the representation extraction layer to obtain the representation of each node. Then, in the intra-view layer, the GAT [152] was applied to update the node representation by capturing the interactions between the atoms of *d_i_*. Moreover, the node representations of drug *d_j_* were extracted and updated in a similar way. Besides, the bipartite graph was fed into the representation extraction layer to extract the representation of each node. Then, in the inter-view layer, the node representation of two drugs were updated by capturing the interactions between the atoms of two drugs via the co-attentional mechanism. The other three DNS encoders were applied to update the output from the previous encoder. Then, the output of the DNS encoder module was input into the self-attention graph (SAG) pooling layer to learn the drug representations and obtain the embedding vectors of *d_i_* and *d_j_*. Finally, the co-attention scoring function was used to calculate the interaction probability of corresponding drug pair based on the embedding vectors.

#### BioDKG-DDI

**Figure 6:**
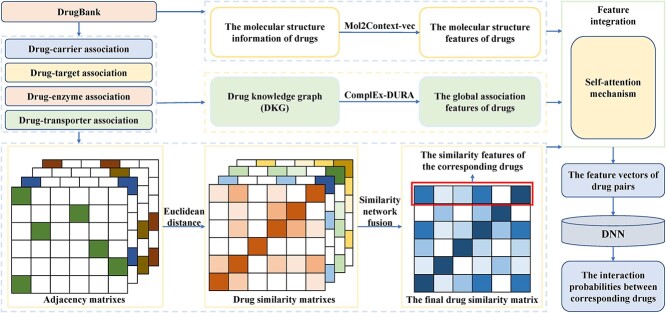
The flowchart of BioDKG-DDI, where the self-attention mechanism is used to fuse the features of two drugs to obtain the features of the corresponding drug pair, which is input into the DNN to obtain the corresponding interaction probability.

Based on the self-attention mechanism [153], Ren *et al.* [154] constructed a model of BioDKG-DDI to predict potential DDIs (Figure 6). Firstly, based on the molecular structure information of drugs recorded in DrugBank [6], a novel molecular representation method named Mol2Context-vec [155] was used to extract the molecular structure features of drugs. Secondly, according to four types of association information (namely, drug–carrier association, drug–enzyme association, drug–target association and drug–transporter association) recorded in DrugBank, they constructed the drug knowledge graph (DKG), where nodes represented biological entities and edges referred to corresponding associations. Then, ComplEx-DURA [156] was applied to extract the global association features of drugs based on the DKG. Thirdly, according to the association information of drug–carrier, the author constructed an adjacency matrix, where rows and columns represent drugs and carriers, respectively. The Euclidean distance between *i*th row and *j*th row of the adjacency matrix was calculated as the similarity between drug *d_i_* and *d_j_*. Then, the similarity matrix based on the association information of drug–carrier was obtained. Besides, the corresponding drug similarity matrices were obtained based on the other three types of associations in a similar way. Then, the similarity network fusion method [157] was used to integrate four similarity matrices to get the final similarity matrix, where each row was regarded as the similarity features of the corresponding drug. Finally, the author used the self-attention mechanism to integrate the above three types of features of each drug to get the final feature and input the final features of the two drugs into the deep neural networks (DNNs) to obtain the interaction probability of the corresponding drug pair.

#### MDF-SA-DDI

**Figure 7:**
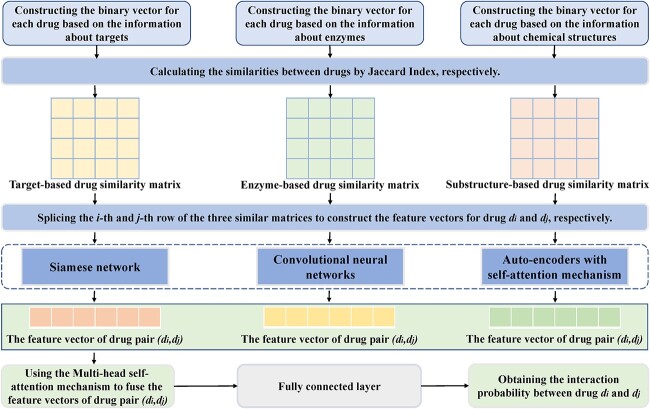
The flowchart of MDF-SA-DDI constructed based on multisource feature fusion and self-attention mechanism.

According to multisource feature fusion and self-attention mechanism [158], Lin *et al.* [159] developed a model named MDF-SA-DDI to predict DDIs (Figure 7). Firstly, for each drug, three binary vectors were constructed based on the information about targets, enzymes and chemical structures of drugs, respectively. Then, based on each type of binary vector, the author calculated the similarity between drugs by Jaccard index and constructed the corresponding similarity matrix. The *i*th row of the three similarity matrices were spliced as the feature vector of drug *d_i_*. Secondly, the authors used the Siamese network [160], convolutional neural networks (CNNs) and autoencoders with self-attention mechanism to fuse the feature vectors of two drugs to obtain the feature vectors of drug pair (*d_i_,d_j_*), respectively. Finally, the multi-head self-attention mechanism was applied to integrate the above three types of feature vectors of drug pair (*d_i_,d_j_*) to obtain the final feature vector, which was input into the full connection layer to calculate the interaction probability between *d_i_* and *d_j_*.

#### Deep feed-forward network-based model

Lee *et al*. [161] proposed a novel DDI prediction model based on autoencoders and the deep feed-forward network. Firstly, the authors calculated three types of drug similarity. Taking drug *d_i_* and *d_j_* as an example, a binary vector was constructed based on the substructure information of each drug, and then the Tanimoto coefficient of the two vectors was calculated as the substructure-based similarity between *d_i_* and *d_j_*. Besides, based on the target genes of drugs, the authors calculated the target gene-based similarity according to the functional interaction (FI) network downloaded from BioGrid [162]:


(60) \begin{equation*} T{S}_{ij}=\frac{\left|\Big\{\left(x,y\right)|x\in{G}_i,y\in{G}_j,d\Big(x,y\left)\le{t}_i\right\}\right|}{\left|\Big\{\left(x,y\right)|x\in{G}_i,y\in{G}_j\Big\}\right|} \end{equation*}

(61) \begin{equation*} {t}_i=\max \left\{d\left(x,y\right)|x,y\in{G}_i\right\} \end{equation*}

where *G_i_* and *G_j_* represent the set of target genes of drug *d_i_* and *d_j_*, respectively, (*x,y*) refers to the gene pair composed of gene *x* and *y*, *d*(*x,y*) is the distance between *x* and *y* in the FI network. In a similar way, the GO term-based drug similarity could be calculated according to the GO term and GO graph [163]. Then, the corresponding similarity matrixes were constructed based on the three types of similarity, respectively. Secondly, for the drug pair (*d_i_,d_j_*), the *i*th and *j*th rows of each similarity matrix were input into the autoencoder to obtain the feature vector of (*d_i_,d_j_*). Finally, the three feature vectors of the drug pair were spliced to obtain the final feature vector, which was input into the deep feed-forward network to obtain the interaction probability between *d_i_* and *d_j_*.

#### R^2^-DDI

Lin *et al.* [164] developed a model relation–aware feature refinement for DDI prediction (R^2^-DDI) to predict potential DDIs. Specifically, for drug *d_i_*, the authors first constructed its molecular graph in the same way as used in SSI-DDI, which was input into DeeperGCN to obtain the graph features vector *E_i_*. The *k*th type of interaction was represented by a learnable vector ${T}_k\in{R}^d$, where *d* represents the dimensions of the interaction feature. Then, in order to construct the relationship among *E_i_*, *E_j_* and *T_k_*, the authors calculated the refinement vectors based on the MLP, which were added to the corresponding original feature vectors to obtain the refined features:


(62) \begin{equation*} {E}_i^{\prime}={E}_i+ MLP\left( concat\left({E}_i,{E}_j,{T}_k\right)\right) \end{equation*}

(63) \begin{equation*} {E}_j^{\prime}={E}_j+ MLP\left( concat\left({E}_i,{E}_j,{T}_k\right)\right) \end{equation*}

(64) \begin{equation*} {T}_k^{\prime}={T}_k+ MLP\left( concat\left({E}_i,{E}_j,{T}_k\right)\right) \end{equation*}

Finally, the probability of the *k*th types of interaction between *d_i_* and *d_j_* was calculated as follows:


(65) \begin{equation*} {P}_{ij}^k= Sigmoid\left({T_k^{\prime}}^T MLP\left( concat\left({E}_i^{\prime},{E}_j^{\prime}\right)\right)\right) \end{equation*}

#### Graph kernel-based approach

In view of the successful application of NLP in biomedicine [165] and computational biology [166], Zhang *et al*. [167] proposed a novel model to detect rapidly accumulating PK DDIs from the biomedical literatures. Unlike the above models of implementing predictions based on the database recording known DDIs, the PK DDI corpus built by Wu *et al.* [168] were employed in this study, which recorded 428 abstracts derived from literatures on PK DDIs. The drug pairs consisting of two drugs appearing in the same sentence were considered as candidate samples. Besides, each sentence with candidate samples was represented by a dependency graph (constructed based on the syntactic structure of sentence) [169] as well as a shallow semantic graph (built according to the shallow semantic relation structure of sentence) [170], respectively. Then, according to the method proposed by Airola *et al*. [171], the authors constructed all-path graph kernels to describe the connections between syntactic and semantic within the sentences. Finally, the graph kernels were used to train the least squares SVM classifier [171], which was applied to identify potential PK DDIs from the literatures.

#### Semantic predication-based model

Based on two widely used NLP tools: MetaMap [172] and SemRep [173], Zhang *et al.* [174] proposed a method to identify potential DDIs via semantic predications. Specifically, firstly, the authors extracted the drug list from clinical data and used MetaMap to map them to the concepts in UMLS [108]. Secondly, from SemMedDB (a database composed of semantic predications generated by SemRep) [175], they extracted four types of semantic predication (namely, drug–predicate–biological function, gene–predicate–biological function, gene–predicate–drug and drug–predicate–gene), where each semantic predication referred to a subject–predicate–object triplet with the UMLS concept as subject and object as well as semantic relationships from the UMLS semantic network as predicates. Thirdly, gene names were normalized to approved gene symbols based on Gene Nomenclature Committee dataset [176]. Fourthly, according to two types of pathway schemas, all drug–drug pairs based on the combinations of semantic predications were collected. In the first schema, drug *d_i_* affects drug *d_j_* through acting on gene *g_k_* (i.e. ${d}_i\to{g}_k\to{d}_j$). In the second schema, *d_i_* affects *g_k1_*, while *d_j_* affects *g_k2_*, where both *g_k1_* and *g_k2_* regulate the same biological function (i.e. ${d}_i\to{g}_{k1}\to biological\ function\leftarrow{g}_{k2}\leftarrow{d}_j$). Finally, the predicted potential DDIs were obtained by filtering out known DDIs from the collected drug–drug pairs.

#### Att-BLSTM

Zheng *et al.* [177] proposed a model named Att-BLSTM to extract DDIs from the biomedical literatures by combining attention mechanism and the recurrent neural network (RNN) with bidirectional long short-term memory (BLSTM). The network architecture of Att-BLSTM was made up of six components, namely, the input layer, embedding layer, input attention layer, merging layer, BLSTM layer and softmax layer. Besides, the DDI-2013 corpus [178] was used in this study, which consisted of the texts describing drugs, and the drug pair in each sentence were manually labeled as either noninteracting or interacting. Firstly, the DDI-2013 corpus was divided into a training set and a test set. For the sentence containing drugs *d_i_* and *d_j_* in the training set, three kinds of information [i.e. the word itself, part of speech (POS), relative distances between the word and each candidate drug in the sentence] of each word were extracted through the input layer, which were encoded into real-valued vectors (i.e. word embedding vectors, POS embedding vectors and position embedding vectors) by the embedding layer through looking up the corresponding embedding dictionary, respectively. Secondly, given that attention mechanisms could be used to quantify the effect of each word on the meaning of the sentence, the input attention layer was used to weigh the word embedding vectors. Thirdly, in the merging layer, each word was represented by a vector obtained by integrating the corresponding three types of embedding vectors. Then, the vectors representing the words were integrated into a sequence of vectors, which was input into the BLSTM layer to learn the global semantic representation of the sentence. Finally, the global representation of sentences was fed into the softmax layer to predict potential DDIs.

#### Position-aware multi-task deep learning method based on BLSTM

To automatically extract DDIs from biomedical texts, Zhou *et al*. [179] developed a position-aware multi-task deep learning method based on BLSTM (PM-BLSTM), whose architecture mainly consisted of four parts: embedding layer, BLSTM layer, position-aware attention layer and multi-task output layer. The DDI-2013 corpus was also used in this study, but unlike in Att-BLSTM where drug pairs were extracted directly from the sentences, for sentences involving more than two drugs, the authors used the rules proposed by Liu *et al.* [180] to filter the drugs to ensure that only one drug pair in each sentence was studied. For the sentence containing drugs *d_i_* and *d_j_*, through a word embedding dictionary and a position embedding dictionary, the embedding layer generated the corresponding embedding vector for each word. Then, the embedding vectors of all words were fed into the BLSTM layer to obtain the matrix composed of the hidden representation of each word. Considering that it was inaccurate to generate attentions only based on the local semantic information for a long sentence, the author utilized the position-aware attention layer to fuse the hidden representations of all words to get the sentence representation. Finally, the sentence representation was fed into the softmax-based classifiers in the multi-task output layer to identify potential DDIs.

#### A two-stage DDIs extraction model

**Figure 8:**
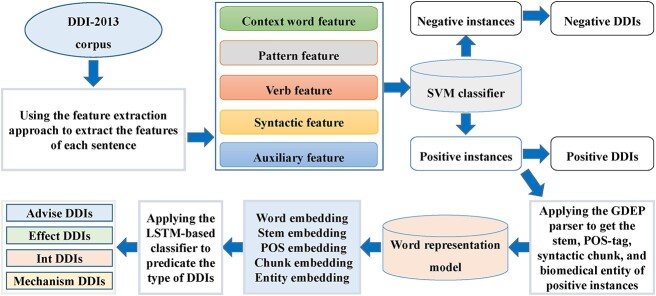
The flowchart of the two-stage DDIs extraction model built based on SVM and LSTM.

Huang *et al*. [181] developed a two-stage method based on SVM and long short-term memory (LSTM) [182] to extract DDIs, where the SVM classifier was applied to identify potential DDIs, while the LSTM-based classifier was used for predicting the type of DDIs, including advise (two drugs are suggested to be taken together in the text), effect (the effects of two drugs taken together are described in the text), mechanism (the pharmacokinetic mechanism of DDI is introduced in the text) and int (there is no additional information about DDI in the text) according to the text description (Figure 8). Specifically, in the first stage, a feature definition approach [183] was used to extract the features for each sentence in the DDI-2013 corpus, including context word feature, pattern feature, verb feature, syntactic feature and auxiliary features. Then, the binary classifier SVM was used to classify the sentences into positive and negative instances. The drug pairs involved in the two instances were regarded as positive DDIs and negative DDIs, respectively. In the second stage, the authors used the GDEP [184] parser to get the stem, POS-tag, syntactic chunk and biomedical entity of positive instances, based on which the word representation model [185] was applied to obtain the word embedding, stem embedding, POS embedding, chunk embedding and entity embedding of each word. Finally, the above embeddings of all words in each positive instance were fed into the LSTM-based classifier to predict the DDI type.

#### Instance position embedding and key external text for DDI extraction

Dou *et al.* [186] developed a framework named instance position embedding and key external text for DDI extraction (IK-DDI), where the instance position embedding was applied to extract DDI information from the DDI Extraction 2013 [187] database, while the key external text describing drugs was derived from the DrugBank. Specifically, firstly, the sentence (containing drugs *d_i_* and *d_j_*) recorded in DDI Extraction 2013 was input into the module (composed of the layers of Embedding, BiLSTM, CNN and MaxPooling) to obtain the feature vector ${f}_{ij}^{\mathrm{int}}$ of drug pair (*d_i_,d_j_*). Secondly, given that the same drug may have different names in different texts, for drug *d_i_* in the drug pair (*d_i_,d_j_*)*,* the authors first calculated the word string similarity *SR_ik_* and word sense similarity *SE_ik_* between the string of *d_i_* and string of drug *d_k_* in DrugBank according to the method presented in the study [188]. Then, the comprehensive similarity was defined:


(66) \begin{equation*} {S}_{ik}=2\times \frac{S{R}_{ik}\times S{E}_{ik}}{S{R}_{ik}+S{E}_{ik}} \end{equation*}

After calculating the comprehensive similarities between *d_i_* and all drugs in DrugBank, the drug with the highest comprehensive similarity with *d_i_* was regarded as the matched drug of *d_i_*. The matched drug of *d_j_* was obtained in a similar way. Next, the authors performed ‘Search key external text’ in DrugBank to mine two key sentences containing the matched drugs of *d_i_* and *d_j_*, respectively, which were input into the module consisting of the layers of Embedding, CNN and MaxPooling to get the feature vector ${f}_{ij}^{ext}$ of drug pair (*d_i_,d_j_*). Then, ${f}_{ij}^{\mathrm{int}}$ and ${f}_{ij}^{ext}$ were input into the fully connected layer to obtain the final feature vector *f_ij_* of the drug pair. Finally, based on *f_ij_*, the softmax classification function was applied to calculate the interaction probability between *d_i_* and *d_j_*.

#### 3D graph and text-based neural network for drug–drug interaction prediction

By integrating the 3D GNN and pretrained text attention mechanism, Chen *et al.* [189] constructed a model named 3D graph and text-based neural network for drug–drug interaction prediction (3DGT-DDI). Firstly, through a force field optimization algorithm called MMFF [190], the authors obtained the 3D structure conformation of drug *d_i_* based on corresponding SMILES, which was fed into the 3D GNN to get the structure-based feature of *d_i_*. Then, the structure-based features of drug *d_i_* and *d_j_* were integrated as the feature vector of drug pair (*d_i_,d_j_*). Secondly, sciBERT, a variant of bidirectional encoder representations from transformers (BERT) pretrained on scientific articles, was applied to tokenize the text describing drug pair (*d_i_,d_j_*) recorded in DDI Extraction 2013. Then, the tokenized text was used as the input of CNN to obtain the text-based feature vector of the drug pair. Finally, the above two types of feature vectors of the drug pair were input into the DNN to obtain the interaction probability between *d_i_* and *d_j_*.

#### Score function-based models

Given the successful application of score function-based models in the field of bioinformatics [42–44, 191], some researchers have developed models based on score functions to identify potential DDIs. The advantages of score function-based models are that the algorithm theory and calculation process involved are relatively easy to understand. Moreover, this type of model does not require negative samples. However, most of the score function-based models make predictions based on known DDIs, so they are not applicable to new drugs. In addition, when using this kind of model to predict DDIs, it is usually necessary to make assumptions about the probability distribution of DDIs, but if the data are inconsistent with the assumptions, the prediction accuracy of the models would be severely affected.

#### Russell–Rao-based model

To predict potential DDIs, Ferdousi *et al*. [192] proposed a computational model based on the Russell–Rao method [193]. To be specific, according to the known associations between drugs and four types of biological elements (including 23 carriers, 115 transporters, 235 enzymes and 1787 targets), the authors constructed four corresponding binary vectors for each drug. Given that there were shared proteins between these four types of biological elements, the authors spliced four types of binary vectors to construct the comprehensive binary vector (2004 dimension) for each drug after removing the redundant proteins. Then, the method of Russell–Rao [193] was used to calculate the interaction probability *P*(*d_i_,d_j_*) between drugs *d_i_* and *d_j_*:


(67) \begin{equation*} P\left({d}_i,{d}_j\right)=\frac{V_{d_i}^T{V}_{d_j}}{2004} \end{equation*}

where ${V}_{d_i}$and ${V}_{d_j}$represent the comprehensive binary vectors of drug *d_i_* and *d_j_*, respectively.

#### Score matrix and PCA-based model

**Figure 9:**
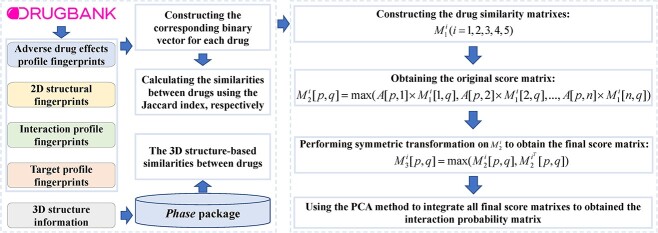
The flowchart of the score matrix and PCA-based model, where the score matrixes are calculated separately based on different similarities, and PCA is used to integrate all the score matrices into the final interaction probability matrix.

Vilar *et al*. [194] proposed a DDIs prediction model by constructing score matrixes according to adjacency matrix and similarity matrixes (Figure 9). Firstly, based on the drug-related information (including 2D structural fingerprints [195], interaction profile fingerprints [196], target profile fingerprints [55] and adverse drug effects (ADEs) profile fingerprints [197]) downloaded from DrugBank, the authors constructed the corresponding binary vector for each drug and then calculated the similarities between drugs using the Jaccard index, respectively. Besides, the authors calculated the 3D structure-based similarities between drugs by the *Phase* package. Then, five corresponding similarity matrixes were constructed, which were represented by ${M}_1^i\left(\mathrm{i}=\mathrm{1,2,3,4,5}\right)$. Secondly, they defined the original score matrix ${M}_2^i$ as follows:


(68) \begin{align*}{M}_2^i\left[p,q\right]=\max (A\left[p,1\right]\times{M}_1^i\left[1,q\right],A\left[p,2\right]\times\nonumber\\{M}_1^i\left[2,q\right],...,A\left[p,n\right]\times{M}_1^i\left[n,q\right]) \end{align*}

where *n* refers to the total number of drugs and *A* represents the adjacency matrix constructed based on known DDIs. Since the matrix ${M}_2^i$ is not symmetric, the authors performed symmetric transformation on the matrix${M}_2^i$ to obtain the final score matrix ${M}_3^i$:


(69) \begin{equation*} {M}_3^i\left[p,q\right]=\max \left({M}_2^i\left[p,q\right],{M}_2^{i^T}\left[p,q\right]\right) \end{equation*}

Finally, the PCA method was used to integrate the score matrixes (i.e. ${M}_3^1,{M}_3^2,{M}_3^3,{M}_3^4,{M}_3^5$) to obtain the interaction probability matrix.

## DISCUSSION AND CONCLUSION

In clinical treatment, in order to cure the disease as soon as possible, patients usually take two or more drugs. The combination of some drugs could not only increase the efficacy of drugs but also delay the emergence of resistance [198]. However, inappropriate drug combinations not only fail to achieve the expected therapeutic effect but also may cause adverse reactions, even toxic reactions. Many drugs are forced to stop selling due to serious adverse reactions caused by DDIs, which not only brings harm to patients but also brings huge economic losses to pharmaceutical companies. More than 20 years ago, the calcium channel blocker mibefradil was withdrawn from the market because it could lead to lethal DDIs by inhibiting the cytochrome P450 3A4 metabolism of certain drugs [199]. For this reason, manufacturers are required to specify DDIs strictly in drug instructions, and consumers must carefully read the instructions when using drugs. As more and more new drugs are approved for clinical treatment, the number of potential DDIs increases rapidly. However, due to the time and money constraints, a large number of potential DDIs that may cause adverse reactions are not provided in drug instructions. With the deepening of the understanding of drug metabolism mechanisms and the rapid accumulation of drug-related data, more and more researchers were committed to building computational models to predict potential DDIs.

In this review, we first introduced the basic conception and classification of DDIs. Some important publicly available databases and web servers about experimentally verified or predicted DDIs were also briefly described. Besides, we summarized three types of prediction models proposed during recent years and discussed the advantages as well as limitations of them. Finally, we pointed out the problems that need to be solved in the future research of DDIs prediction and provided corresponding suggestions. In general, this review is helpful for researchers to have a comprehensive understanding of DDIs prediction and provides valuable guidance for their research studies, especially in the construction of models; they can weigh the advantages and disadvantages of various models to build the most suitable model for their own research studies.

Several researchers have written reviews to summarize the DDIs prediction models. For example, Zhang *et al.* [200] summarized deep learning-based models for extracting DDIs from the literatures and divided them into three categories: CNN-based model, RNN-based model and recursive NN-based model. Then, the authors compared the performance of these models in the DDI corpus and summarized the strengths as well as weaknesses of these models. Finally, the authors discussed the challenges and future prospects of extracting DDIs by deep learning-based models. Besides, Lin *et al.* [201] first listed the DDIs prediction models constructed based on deep learning as well as graph learning and evaluated their performance based on different tasks, including the binary classification task, multi-class classification task as well as multi-label classification task. In addition, they introduced a variety of molecular representation methods of drugs, such as sequence-based, 2D graph-based, 3D graph-based, knowledge graph-based and so on. Finally, the authors discussed the potential technical challenges and highlighted the future directions of predicting DDIs by the above two types of models.

Although both reviews provided detailed summaries of models for DDIs prediction, they mainly focused on deep learning-based models. There were other types of models that were built to predict potential DDIs, such as traditional machine learning-based models and score function-based models. In order to enable researchers to have a more comprehensive understanding of the research studies related to DDIs prediction, we summarized the above three types of models in this review.

There were obvious differences among the above three types of models. For example, the traditional machine learning-based models were designed to leverage classical machine learning algorithms to make reliable predictions by building efficient features or solving specific optimization problems. The deep learning-based models were designed to automatically learn the significant features of drug pairs and then implement DDIs prediction. In the score function-based models, the authors constructed the corresponding function based on probability distribution or statistical analysis to calculate the interaction probabilities between drugs. Besides, different from the other two types of models used to implement DDIs prediction based only on the data about drug similarity and known DDIs, most deep learning-based models also utilized substructure information about drugs, and the models built based on NLP methods were applied to mine potential DDIs based on the texts in the literatures. Moreover, in the deep learning-based models, some specified modules (including self-attention mechanism, Siamese network and so on) were used to fuse the features of two drugs to obtain the features of the corresponding drug pair, rather than splicing the features of two drugs directly in the other two types of models. Compared with the other two types of models, the score function-based models were easier to understand and does not require negative samples. We briefly summarized each type of model below.

A variety of classical algorithms were involved in the traditional machine learning-based models, such as label propagation, MRFs, random forest, logistic regression, SVM, matrix factorization, ensemble learning and so on. For example, in INDI [57], the authors first calculated seven types of drug similarities and obtained the scores between two drugs through a scoring scheme based on the drug similarities. Then, according to the scores between two drugs, the feature vector of corresponding drug pair was constructed, which was used as the input of logistic regression classifier to implement DDIs predictions. In the DDIs prediction model built by Liu *et al.* [73] based on random forest, the authors first constructed the feature vector for each drug pair according to the information about the chemical structure and target protein of drugs. Then, the feature vector was fed into the random forest model to calculate the interaction probability between the corresponding drugs.

In the deep learning-based model, researchers applied different techniques (i.e. CNNs, DNNs, GNNs, Graph Embedding and so on) to predict DDIs based on drug-related information or used relevant methods of NLP to mine potential DDIs from texts. Taking MCFF-MTDDI [146] as an example, after obtaining the chemical structure-based feature and KG fusion representations of each drug pairs, the two types of features were input into a GRU-based multichannel feature fusion framework to obtain the fused feature vector, which was used as the input of the multi-class classifier to implement the multi-class classification tasks. Besides, the extra label-based feature vector and fused feature vector of drug pair were concatenated and input into the multi-label classifier to implement the multi-label classification. In the model constructed by Huang *et al.* [181] based on the two-stage method, after defining the features of each sentence, the authors first divided the sentences into positive instances and negative instances using SVM. Then, they used the word representation model to obtain multiple embedding of each word in the positive instances, which was fed into the LSTM-based classifier to predict the type of the corresponding DDIs.

In the score function-based models, the authors defined score functions from different perspectives to calculate the interaction probabilities between drugs. For example, in the model constructed based on the Russell–Rao method, according to four kinds of association information about drugs, the authors constructed corresponding binary vectors for each drug, which were integrated into a comprehensive binary vector. Finally, the Russell–Rao method was used to calculate the interaction probability based on the comprehensive binary vectors of two drugs.

Next, we summarized the advantages and disadvantages of the above three types of models. The traditional machine learning-based model can be used to make large-scale rapid prediction for potential DDIs. Besides, the main advantage of this type of model is that they are suitable for new drugs, such as TMFUF [99], which can be used to predict DDIs between new drugs. However, they still have some limitations. For example, the traditional machine learning-based models involve multiple parameters, and the setting of parameter values limits the model performance to some extent. Besides, researchers tended to define the feature vectors of drug pairs based on the similarities between drug, so constructing feature vectors with higher significance is still an urgent problem to be solved. Moreover, given that negative samples were extremely difficult to obtain, researchers treated candidate samples as negative samples to train this type of models, which limited the performance of the model to some extent. Compared with traditional machine learning methods, deep learning methods can automatically mine the significant features of drugs. In addition, deep learning-based models have high flexibility in feature fusion. For instance, in MDF-SA-DDI [159], the self-attention mechanism was used to fuse the feature vectors of drug pairs, while a GRU-based multichannel feature fusion framework was applied to integrate the feature vectors of drug pairs in MCFF-MTDDI [146]. Compared with the simple splicing of feature vectors, the above fusion methods can fully fuse features. However, implementing predictions with deep learning-based models often takes more time. As with models constructed based on the traditional machine learning algorithms, the scarcity of reliable negative samples severely limits the performance of models. Besides, deep learning-based models lack interpretability. The advantage of score function-based models is that the algorithm theory and calculation process involved are relatively easy to understand. Moreover, this type of model does not require negative samples. However, they still have some deficiencies. For instance, most of the score function-based models are not suitable for new drugs. In addition, when these models are applied to predict potential DDIs, assumptions about the probability distribution are often required, but if the data are inconsistent with the assumptions, the prediction accuracy of the models will be severely affected.

Considering the advantages and disadvantages of each type of models, in my opinion, it is best for researchers to build models based on deep learning algorithms in the future research. Compared with the other two types of models, the computational efficiency of deep learning-based models is lower, but their accuracy is generally higher. Besides, deep learning-based models can predict interactions between drug substructures, which is beneficial for us to understand the mechanism of DDIs. NLP models could not only be used to mine a large number of DDIs from the literatures but also help researchers learn detailed information about DDIs. As for the disadvantages of this type of model, instead of randomly selecting drug pairs without known interactions as negative samples, designing methods to identify reliable negative samples is more conducive to further improving the accuracy of the models. Given the lack of interpretability in most deep learning-based models, it is necessary to perform interpretability analysis on these models. In addition, in view of the fact that the values of parameters in models affect the prediction performance to a certain extent, designing the model to determine the optimal values of the parameters helps further improve the prediction accuracy.

To evaluate the predictive performance of computational models, most researchers conducted k-fold cross validation and case studies. Besides, there are other methods used to evaluate performance. For example, in MCFF-MTDDI [146], Chen *et al.* applied seven classical evaluation indicators (including Accuracy, Macro-Precision, Macro-Recall, Macro-F1, Cohen’s Kappa, AUC and AUPR) to evaluate the prediction performance of model in a multi-class classification task, while AUC and AUPR were used to evaluate the performance of model in multi-label prediction tasks. In BioDKG-DDI [154], the Matthews’s correlation coefficient (MCC) was used to evaluate the effectiveness of the model. These indicators are very convincing in assessing the performance of DDIs prediction models. Besides, some researchers performed ablation studies to assess the effect of each module on predictive performance. For example, in SSI-DDI [129], the authors removed the co-attention layer and changed the number of GAT layers to evaluate the effect of the corresponding two modules on the prediction accuracy, respectively.

In view of that discovering potential DDIs would be beneficial to drug development and clinical treatment, researchers have developed several DDI prediction models with superior performance. However, there are still some problems to be solved in the future. Firstly, the data are extremely unbalanced, i.e. the number of positive samples is much smaller than the number of candidate samples. Therefore, it is necessary to collect more drug–drug pairs with known interactions as positive samples in the future work. Secondly, current research studies focus on the identification of potential DDIs and the prediction of DDIs types, but less research has been done on the severity of DDIs. Besides, the existing prediction models do not take into account the effect of drug dose on DDIs. Thirdly, current models can only be used to predict interactions between two drugs, which is far from enough. In clinical treatment, patients often need to take more than two drugs. Therefore, it is of great significance to study the interactions among multiple drugs. In order to solve the above two problems, it is necessary to build the corresponding databases to provide the data foundation for the subsequent research studies. Fourthly, as more and more drug-related data are generated, applying deep learning algorithms to effectively merge them is expected to further improve the prediction accuracy. Finally, many models made predictions only based on the similarity and association information of drugs, but these models could not reveal the mechanism of interactions. If text information describing the drugs is introduced, this problem is expected to be improved.

**Key Points**

- We introduced the basic conception and classification of DDIs. In addition, several databases and web servers about DDIs were introduced.
- Paying attention to DDIs is of great significance to adopt effective combination therapy and further improve the quality of medical treatment.
- Revealing DDIs through experiments is extremely time-consuming and costly, and building the computational models to calculate the interaction probabilities between drugs could be an important complement to experimental methods.
- Based on the calculation principle of the models, we simply divided the models into three categories: traditional machine learning-based models, deep learning-based models and score function-based models.
- We briefly discussed the advantages and limitations of existing computational models and put forward the existing problems in the current DDIs prediction research, which need to be resolved in the future work.

## Data Availability

The source code and data of Bayesian probabilistic method-based model are available at http://www.picb.ac.cn/hanlab/DDI. The source code and data of LCM-DS are available at https://github.com/JustinShi2016/ScientificReports2018. The source code and data of Network algorithm and matrix perturbation algorithm-based model are available at https://github.com/zw9977129/drug-drug-interaction/. The source code and data of SFLLN are available at https://github.com/BioMedicalBigDataMiningLabWhu/SFLLN. The source code and data of DDIMDL are available at https://github.com/YifanDengWHU/DDIMDL. The source code and data of SSI-DDI are available at https://github.com/kanz76/SSI-DDI. The source code and data of STNN-DDI are available at https://github.com/zsy-9/STNN-DDI. The source code and data of META-DDIE are available at https://github.com/YifanDengWHU/META-DDIE. The source code and data of DANN-DDI are available at https://github.com/naodandandan/DANN-DDI. The source code and data of MRCGNN are available at https://github.com/Zhankun-Xiong/MRCGNN. The source code and data of MCFF-MTDDI are available at https://github.com/ChendiHan111/MCFF-MTDDI. The source code and data of DSN-DDI are available at https://github.com/microsoft/Drug-Interaction-Research/tree/DSN-DDI for-DDI-Prediction. The source code and data of MDF-SA-DDI are available at https://github.com/ShenggengLin/MDF-SA-DDI. The source code and data of R2-DDI are available at https://github.com/linjc16/R2-DDI. The source code and data of Graph kernel-based approach are available at https://sbmi.uth.edu/ccb/resources/ddi.htm. The source code and data of IK-DDI are available at https://github.com/DouMingLiang/IK-DDI. The source code and data of 3DGT-DDI are available at https://github.com/hehh77/3DGT-DDI.

## Author Biographies

**Yan Zhao** is a PhD student of School of Information and Control Engineering, China University of Mining and Technology. His research interests include bioinformatics, complex network algorithm and machine learning.

**Jun Yin** is a full-time master student of School of Information and Control Engineering, China University of Mining and Technology. His research interests include bioinformatics, complex network algorithm and machine learning.

**Li Zhang** is a PhD student of School of Information and Control Engineering, China University of Mining and Technology. His research interests include bioinformatics, drug discovery and the graph neural network.

**Yong Zhang**, PhD, is a professor of School of Information and Control Engineering, China University of Mining and Technology. His research interests include swarm intelligence, pattern recognition and machine learning.

**Xing Chen**, PhD, is a professor of School of Science, Jiangnan University. His research interests include complex disease-related non-coding RNA biomarker prediction, computational models for drug discovery and early detection of human complex disease based on big data and artificial intelligence algorithms.
